# Phosphotyrosine-Mediated Regulation of Enterohemorrhagic *Escherichia coli* Virulence

**DOI:** 10.1128/mBio.00097-18

**Published:** 2018-02-27

**Authors:** Colin D. Robertson, Tracy H. Hazen, James B. Kaper, David A. Rasko, Anne-Marie Hansen

**Affiliations:** a Department of Microbiology and Immunology, University of Maryland School of Medicine, Baltimore, Maryland, USA; b Institute for Genome Sciences, University of Maryland School of Medicine, Baltimore, Maryland, USA; UCLA School of Medicine

**Keywords:** EHEC, T3SS, gene regulation, tyrosine phosphorylation

## Abstract

Enteric pathogens with low infectious doses rely on the ability to orchestrate the expression of virulence and metabolism-associated genes in response to environmental cues for successful infection. Accordingly, the human pathogen enterohemorrhagic *Escherichia coli* (EHEC) employs a complex multifaceted regulatory network to link the expression of type III secretion system (T3SS) components to nutrient availability. While phosphorylation of histidine and aspartate residues on two-component system response regulators is recognized as an integral part of bacterial signaling, the involvement of phosphotyrosine-mediated control is minimally explored in Gram-negative pathogens. Our recent phosphotyrosine profiling study of *E. coli* identified 342 phosphorylated proteins, indicating that phosphotyrosine modifications in bacteria are more prevalent than previously anticipated. The present study demonstrates that tyrosine phosphorylation of a metabolite-responsive LacI/GalR family regulator, Cra, negatively affects T3SS expression under glycolytic conditions that are typical for the colonic lumen environment where production of the T3SS is unnecessary. Our data suggest that Cra phosphorylation affects T3SS expression by modulating the expression of *ler*, which encodes the major activator of EHEC virulence gene expression. Phosphorylation of the Cra Y47 residue diminishes DNA binding to fine-tune the expression of virulence-associated genes, including those of the locus of enterocyte effacement pathogenicity island that encode the T3SS, and thereby negatively affects the formation of attaching and effacing lesions. Our data indicate that tyrosine phosphorylation provides an additional mechanism to control the DNA binding of Cra and other LacI/GalR family regulators, including LacI and PurR. This study describes an initial effort to unravel the role of global phosphotyrosine signaling in the control of EHEC virulence potential.

## IMPORTANCE

Enterohemorrhagic *Escherichia coli* (EHEC) causes outbreaks of hemorrhagic colitis and the potentially fatal hemolytic-uremic syndrome. Successful host colonization by EHEC relies on the ability to coordinate the expression of virulence factors in response to environmental cues. A complex network that integrates environmental signals at multiple regulatory levels tightly controls virulence gene expression. We demonstrate that EHEC utilizes a previously uncharacterized phosphotyrosine signaling pathway through Cra to fine-tune the expression of virulence-associated genes to effectively control T3SS production. This study demonstrates that tyrosine phosphorylation negatively affects the DNA-binding capacity of Cra, which affects the expression of genes related to virulence and metabolism. We demonstrate for the first time that phosphotyrosine-mediated control affects global transcription in EHEC. Our data provide insight into a hitherto unexplored regulatory level of the global network controlling EHEC virulence gene expression.

## INTRODUCTION

The enteric human pathogen enterohemorrhagic *Escherichia coli* (EHEC) causes food-borne outbreaks of hemorrhagic colitis and the potentially fatal hemolytic-uremic syndrome worldwide (1–4). EHEC is a challenge to control epidemiologically because it has a low infectious dose (5). EHEC infection is characterized by the formation of intestinal attaching and effacing (A/E) lesions due to the activity of a type III secretion system (T3SS) (6–8). The locus of enterocyte effacement (LEE) pathogenicity island (PAI) contains five major operons that encode components of the T3SS, the adhesin intimin, the translocated intimin receptor (Tir), effector proteins, and transcriptional regulators (6, 9). The *LEE1* operon encodes the regulator Ler, which is a major activator of virulence-associated genes located within and outside the LEE island (10, 11). To ensure appropriate production of the T3SS, *LEE* expression is tightly controlled and linked to environmental cues such as nutrient availability. The coordinated regulation of genes involved in virulence and metabolism is orchestrated by a multifaceted regulatory network that integrates environmental cues to ensure the optimal temporal-spatial expression of genes, a requirement for successful infection by EHEC (10, 12–14).

Signaling by two-component systems involving phosphorylation of histidine and/or aspartate is a well-characterized central regulatory mechanism known to control the virulence potential of EHEC (15–17). Yet, the involvement of protein tyrosine phosphorylation as a global regulatory mechanism is an understudied aspect of prokaryotic signaling, which is in contrast to its fundamental role in eukaryotes (18). Phosphotyrosine-mediated regulation is a dynamic regulatory process that relies on the activities of the tyrosine kinase(s) and the cognate tyrosine phosphatase(s) (19). Given that bacterial tyrosine kinases phosphorylate their target proteins less efficiently than two-component system kinases because of relaxed substrate specificity, tyrosine phosphorylation provides a fine-tuning response rather than eliciting an on-off response as described for regulation by traditional two-component system kinases (20). The two currently known *E. coli* tyrosine kinases (Etk and Wzc) are associated with exopolysaccharide synthesis, antibiotic resistance, phage lysogenization, and heat shock response and also affect EHEC virulence by regulating group 4 capsule synthesis (21–26). However, research elucidating the role of phosphotyrosine signaling in global regulation has been limited by the relatively low number of tyrosine-phosphorylated proteins previously known (about 32 in *E. coli*). Our recent phosphotyrosine profiling study of *E. coli* (*E. coli* K-12 and EHEC), as well as a subsequent study involving *Shigella flexneri*, revealed tyrosine phosphorylation of between 4 and 12% of the proteomes, indicating that the prevalence of phosphotyrosine modifications is even higher than in eukaryotic cells, where about 2% of the proteome is tyrosine phosphorylated (27–29). These findings refute the previous notion that tyrosine phosphorylation occurs primarily in eukaryotes. Of the 512 phosphotyrosine sites on 342 proteins combined in *E. coli* K-12 and EHEC O157:H7 that we identified by a mass spectrometry-based phosphoproteomic approach, most relate to fundamental cell functions and virulence, indicating a central regulatory role of tyrosine phosphorylation in *E. coli* (27). Interestingly, we identified phosphotyrosine modifications on nine global transcriptional regulators associated with *LEE* expression (27), suggesting that phosphotyrosine signaling could play an important role in the control of EHEC virulence potential. Indeed, we previously demonstrated that tyrosine phosphorylation of the regulator SspA positively affects the production and secretion of *LEE*-encoded T3SS proteins and is required for optimal A/E lesion formation by EHEC (27). The proteins identified as being tyrosine phosphorylated also included the sugar-sensing LacI/GalR family transcriptional regulator Cra, which is required for LEE expression and A/E lesion formation under gluconeogenic conditions (30, 31).

The ability of enteric pathogens to effectively link metabolism to virulence by sensing compounds produced by the host and the resident microbiota is paramount for their survival and successful colonization of the gastrointestinal tract (12, 32–36). The EHEC colonization site at the colonic epithelium comprises an aerobic environment that is rich in gluconeogenic carbon sources derived from the breakdown of host carbohydrates by the microbiota (34, 37, 38). Cra facilitates the ability of EHEC to switch effectively between the use of glycolytic carbon sources and that of gluconeogenic carbon sources, which provides a competitive advantage over resident *E. coli* in mouse and bovine infection models (39–44). In particular, Cra is a key player in the control of the carbon flux in central metabolic pathways in response to carbon source availability by coordinating the expression of genes involved in glycolysis and gluconeogenesis (30, 45–49). Cra activity is regulated in a glycolytic flux-dependent manner through binding of the glycolytic metabolites fructose-1,6-bisphosphate (FBP) and fructose-1-phosphate, inducing conformational changes that diminish Cra DNA-binding ability under glycolytic conditions (30, 46, 47, 50, 51). A comparison of the global transcriptomes of wild-type EHEC and a *cra* deletion mutant demonstrated that Cra is a global regulator in EHEC that affects the expression of genes associated with virulence and major carbon metabolic pathways (52). Cra links metabolism to EHEC virulence independently or in concert with the response regulator KdpE by controlling the expression of virulence genes, including *LEE1/ler*, to induce the production of the T3SS under gluconeogenic conditions, thought to represent the environment at the colonization site (31, 52, 53). On the contrary, *LEE* expression, and thereby redundant production of the T3SS, is prevented in the glycolytic lumen environment. Hence, coordinated regulation of virulence and metabolic genes by Cra in response to carbon source availability promotes survival and successful host colonization. Cra activation of *LEE* expression was also recently demonstrated to depend on oxygen availability (53). However, it is currently unknown whether Cra activity is directly regulated by posttranslational modification mechanisms other than carbon catabolite repression such as protein phosphorylation. Notably, our phosphotyrosine profiling study identified phosphorylation of the Cra Y47 residue in an EHEC O157:H7 isolate grown under glycolytic conditions (27). Cra Y47 is a highly conserved and functionally important residue in LacI/GalR family proteins, where it is located in the linker region next to the DNA recognition helix of the helix-turn-helix (HTH) motif in the N-terminal DNA-binding domain (54–56) (see Fig. S1 in the supplemental material). Structural studies of LacI/GalR family regulators bound to operator DNA demonstrate that the tyrosine residues corresponding to Cra Y47 make base-specific contact with the DNA backbone through hydrogen bond formation (57–60). Thus, we hypothesize that the introduction of a negative charge through phosphorylation of Y47 could affect Cra DNA binding and, with that, EHEC virulence gene expression.

10.1128/mBio.00097-18.2 FIG S1  The phosphorylated Cra Y47 residue is located in the DNA-binding domain of LacI/GalR family regulators. Dimeric structure of LacI bound to *lac* operator DNA indicating the conserved residue Y47 that is phosphorylated in Cra. The tetramerization domain (I), regulatory ligand-binding domain (II), linker region (III), and DNA-binding domain (IV) are shown in a blue ribbon structure, and DNA is gray (right panel). The magnified structure of the linker region and DNA-binding domain is shown on the right with Y47 in green. The hydroxyl group of Y47 phosphorylated in Cra is shown in red. The structure of LacI bound to *lac* O1O3 (PDB code 1EFA) was visualized with PyMol (Schrödinger, LLC). Download FIG S1, TIF file, 0.5 MB. Copyright © 2018 Robertson et al. 2018 Robertson et al. This content is distributed under the terms of the Creative Commons Attribution 4.0 International license.

The present study demonstrates that phosphorylation of Cra Y47 negatively affects production of the T3SS and A/E lesion formation under glycolytic conditions, thereby controlling EHEC virulence. Global transcriptome analysis reveals that phosphotyrosine-mediated regulation by Cra affects the transcription of genes involved in both virulence and metabolism. In particular, Cra phosphorylation negatively affects the expression of *LEE* genes that encode the T3SS. This study demonstrates that tyrosine phosphorylation diminishes the DNA-binding capacity of Cra, indicating that phosphotyrosine-mediated regulation provides an additional mechanism to regulate Cra activity besides catabolite-mediated allosteric control. Our data further suggest that tyrosine phosphorylation could serve as a general mechanism to control the DNA binding of LacI/GalR family regulators in addition to ligand-mediated regulation.

## RESULTS

### Phosphorylation of Cra Y47 negatively affects the production and secretion of LEE-encoded T3SS proteins.

**FIG 1:**
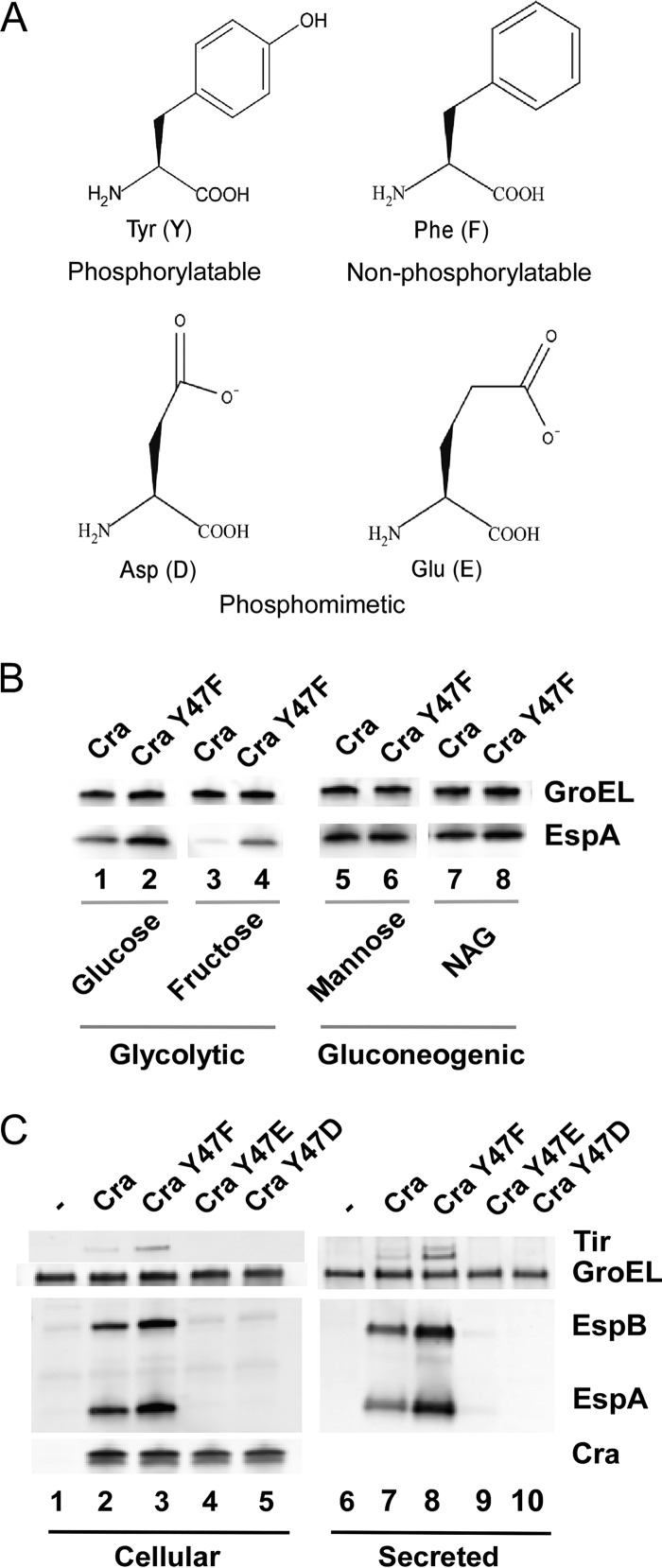
Phosphorylation of Cra Y47 negatively affects the production and secretion of T3SS proteins. (A) Structure of amino acid residues used for substitution of Y47 to generate nonphosphorylatable and phosphomimetic Cra. (B) Cra Y47 affects the production of the T3SS protein EspA under glycolytic conditions. Western analysis of EHEC expressing wild-type Cra and nonphosphorylatable Cra Y47F in M9 medium supplemented with 0.4% glycolytic (lanes 1 to 4) or gluconeogenic (lanes 5 to 8) carbon sources as indicated. NAG is *N*-acetylglucosamine. EspA and GroEL were detected by Western analysis. (C) Cra Y47 affects T3SS protein production and secretion under glycolytic conditions (M9 with 0.4% glucose). The abundance of *LEE*-encoded proteins in cell lysates (lanes 1 to 5) and culture supernatants (lanes 6 to 10) of an EHEC *cra* deletion-containing strain producing wild-type Cra, nonphosphorylatable Cra (Y47F), and phosphomimetic Cra (Y47D and Y47E) variants was determined by Western analysis. GroEL served as an internal control for the total cellular protein loaded and was added to culture supernatants as a control for protein precipitation.

To determine the functional importance of Cra Y47 phosphorylation, we constructed two classes of Cra Y47 substitution variants, a nonphosphorylatable phenylalanine substitution Cra variant (Y47F) and a phosphomimetic Cra variant that has Y47 replaced with a negatively charged aspartate (Y47D) or glutamate (Y47E) residue (Fig. 1A), which is traditionally used to generate a derivative that mimics constitutively phosphorylated protein. To assess whether phosphotyrosine-mediated regulation by Cra occurs under glycolytic conditions where Cra Y47 was identified as phosphorylated (27), we compared the abilities of plasmid-encoded wild-type Cra and nonphosphorylatable Cra Y47F to complement T3SS expression in a *cra* deletion-containing variant of the EHEC EDL933 Δ*stx* mutant strain, TUV93-0 (61), grown in M9 medium with a 0.4% concentration of glycolytic or gluconeogenic carbon sources. We verified that expression of Cra from a low-copy-number plasmid supports the production and secretion of T3SS proteins, as well as A/E lesion formation (*P* = 0.36), in a manner similar to that of chromosome-encoded Cra, thereby ruling out any gene dosage effect from expressing plasmid-encoded Cra (Fig. S2A and B). EHEC producing nonphosphorylatable Y47F Cra versus wild-type Cra under glycolytic conditions (glucose and fructose) showed a 2-fold increased abundance of the *LEE*-encoded T3SS protein EspA (Fig. 1B, compare lanes 2 and 4 with lanes 1 and 3), whereas growth on gluconeogenic carbon sources (mannose and *N*-acetylglucosamine) diminished the regulatory impact of Cra Y47 (Fig. 1B, compare lanes 6 and 8 with lanes 5 and 7). Thus, Cra Y47 negatively affects T3SS protein production in response to glycolytic conditions, representing the carbon source status of the large intestine lumen where T3SS expression is unnecessary. Under glycolytic conditions, EHEC producing nonphosphorylatable Cra Y47F exhibited increased the production and secretion of *LEE*-encoded T3SS proteins EspA, EspB, and Tir by 2- to 4-fold compared to that of the wild type (Fig. 1C, compare lane 3 with lane 2 and lane 8 with lane 7), suggesting a negative regulatory effect of Cra Y47. EHEC expressing the phosphomimetic Y47D and Y47E Cra variants demonstrated diminished T3SS protein production and secretion (Fig. 1C, lanes 4 to 5 and 9 to 10), suggesting that phosphorylation of Cra Y47 negatively affects *LEE* expression.

10.1128/mBio.00097-18.3 FIG S2  Cra regulation of EHEC virulence. (A) Chromosomal and plasmid-encoded Cra proteins support the production and secretion of the T3SS proteins EspA and EspB in a similar manner. Western analysis of EHEC TUV93-0 producing chromosome-encoded Cra (lane 1) and an isogenic *cra* deletion derivative producing plasmid-encoded Cra (lane 2) in M9 medium supplemented with 0.4% glucose is shown. The presence of cellular Cra, EspA, and EspB, as well as secreted EspA and EspB, was detected as indicated. GroEL served as a loading control. (B) EHEC producing chromosomal Cra (column 1) or plasmid-encoded Cra (column 2) exhibits similar A/E lesion phenotypes. A/E lesion formation efficiency was determined by the number of clusters containing at least eight lesions per 100 HeLa cells (*n* = 300). Standard deviations are indicated. The unpaired *t* test was used to determine statistically significant differences by using a threshold *P* value of <0.05. (C) Cra Y47 regulation of EHEC virulence gene expression. Quantitative real-time PCR measured *LEE* (*ler/LEE1*, *sepZ/LEE2*, *escV/LEE3*, and *espB/LEE4*), *nleA*, and *stcE* transcript levels in EHEC producing nonphosphorylatable Cra Y47F (gray bars) and phosphomimetic Cra Y47D (black bars) relative to those in wild-type Cra. Cells were grown aerobically to early stationary phase in M9 medium supplemented with 0.4% glucose. Data represent the mean relative fold expression values and standard deviations of triplicate experiments. An asterisk indicates a statistically significant difference from transcript levels in cells producing wild-type Cra as determined by the unpaired *t* test (*P* < 0.05). Download FIG S2, TIF file, 0.5 MB. Copyright © 2018 Robertson et al. 2018 Robertson et al. This content is distributed under the terms of the Creative Commons Attribution 4.0 International license.

### Phosphotyrosine-mediated regulation by Cra affects global transcription.

**FIG 2:**
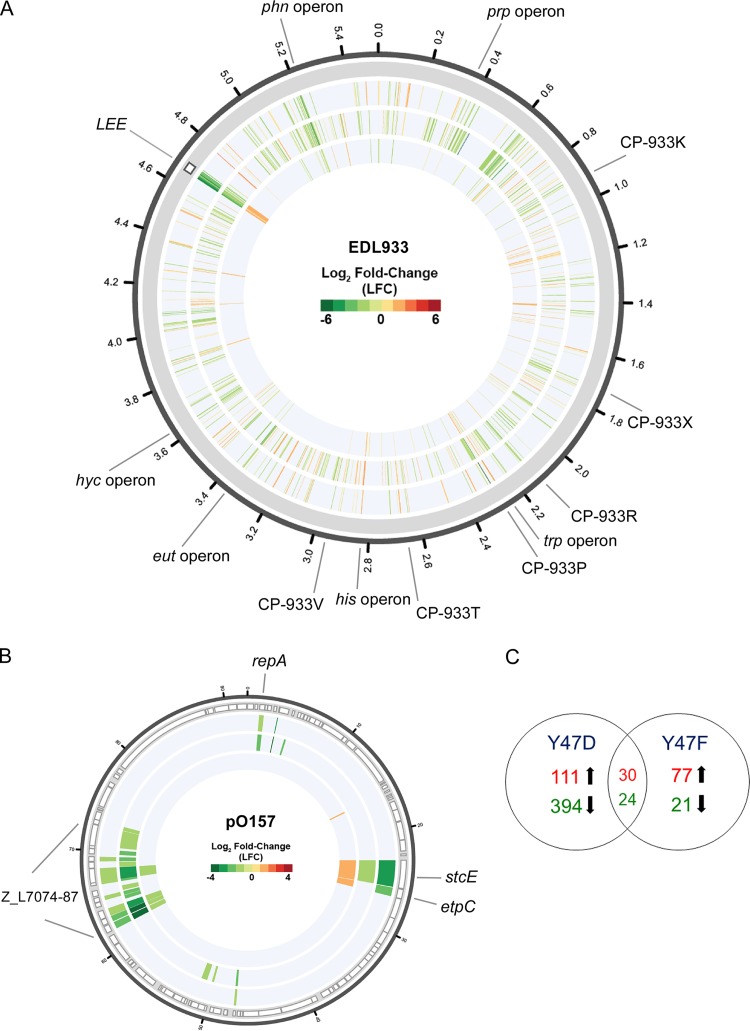
Phosphotyrosine-mediated regulation by Cra affects global transcription. Comparative RNA-Seq transcriptome analyses of EHEC expressing wild-type Cra, nonphosphorylatable Cra (Y47F), and phosphomimetic Cra (Y47D) variants in M9 medium containing 0.4% glucose. Shown are circular plots of the LFCs in gene expression from the chromosome (A) and virulence plasmid pO157 (B). Genes are organized clockwise on the basis of their locus tags for the chromosome and pO157. The tracks located on the circular plots represent transcriptome comparisons of EHEC expressing Cra Y47F versus wild-type Cra (inner track), Cra Y47D versus wild-type Cra (mid track), and Cra Y47D versus cra Y47F (outer track). The tracks display the LFCs of each gene with significant DE based on the criteria LFC ≥1 and ≤1. The locations of the LEE island and selected DE loci are indicated. Red indicates increased DE, green indicates decreased DE, and white indicates no significant difference in expression. (C) Venn diagram illustrating the numbers of shared and unique DE genes of EHEC producing nonphosphorylatable (Y47F) and phosphomimetic (Y47D) Cra compared to wild-type Cra. The numbers of genes with differentially increased (red) and decreased (green) expression are shown.

Given that the Cra Y47 residue is functionally important, we assessed the global regulatory effect of Cra tyrosine phosphorylation by defining the global transcriptomes of EHEC producing wild-type Cra, nonphosphorylatable Cra Y47F, and phosphomimetic Cra Y47D by transcriptome sequencing (RNA-Seq) analysis. We determined the global transcriptomes of cells grown to early stationary phase under glycolytic conditions (0.4% glucose), where Cra Y47 affects the production and secretion of T3SS proteins (Fig. 1B and C). Under these conditions, we expected to specifically identify genes that respond to phosphotyrosine-mediated regulation by Cra Y47 in addition to metabolite-mediated control. The three RNA samples, each prepared in duplicate, generated between 39.5 million and 70.9 million Illumina HiSeq reads per sample (a total of approximately 339.3 million reads), which were mapped to the corresponding genome or virulence plasmid pO157 (Table S1). We compared the transcriptomes and considered genes significantly differentially expressed (DE) if the transcript level log_2_ fold change (LFC) was ≥1 or ≤−1 (*P* ≤ 0.01). Comparison of the global transcriptomes of EHEC producing wild-type Cra, Cra Y47F, and Cra Y47D identified a total of 691 DE genes located on the chromosome or plasmid pO157 (Fig. 2A and B; Table S2). Of note, there is a chromosome-wide distribution of the genes, indicating that phosphotyrosine-mediated gene regulation was not limited to only a small region of the chromosome, whereas the DE genes on the pO157 plasmid are clustered into a few regions associated with plasmid replication and a type II secretion system (T2SS).

10.1128/mBio.00097-18.6 TABLE S1  RNA-Seq samples analyzed in this study. Download TABLE S1, XLSX file, 0.01 MB. Copyright © 2018 Robertson et al. 2018 Robertson et al. This content is distributed under the terms of the Creative Commons Attribution 4.0 International license.

10.1128/mBio.00097-18.7 TABLE S2  DE genes in EHEC expressing wild-type Cra, Cra Y47F, and Cra Y47D. Download TABLE S2, XLS file, 0.2 MB. Copyright © 2018 Robertson et al. 2018 Robertson et al. This content is distributed under the terms of the Creative Commons Attribution 4.0 International license.

Comparison of the global transcriptomes of EHEC producing phosphomimetic Cra Y47D versus wild-type Cra identified 559 DE genes (141 increased and 418 decreased), whereas EHEC producing nonphosphorylatable Cra Y47F versus wild-type Cra identified 152 DE genes (107 increased and 45 decreased) (Table S3). Accordingly, comparison of transcriptomes for EHEC producing phosphomimetic Cra Y47D with nonphosphorylatable Cra Y47F identified a total of 304 DE genes with 219 being decreased and 85 being increased (Table S3). To identify DE genes that were unique to the transcriptomes for EHEC producing Cra Y47D or Cra Y47F, we excluded 54 genes that demonstrated DE in both transcriptomes compared to that of cells producing wild-type Cra. This comparison revealed that EHEC producing phosphomimetic Cra Y47D expresses 505 DE genes that are subject to phosphotyrosine-mediated regulation, which is a 5-fold greater number than the 98 DE genes identified in EHEC producing nonphosphorylatable Cra Y47F (Fig. 2C; Table S3). Thus, Cra tyrosine phosphorylation exhibits an overall negative regulatory effect on global transcription. The lower number of DE genes identified for EHEC producing nonphosphorylatable Cra Y47F than phosphomimetic Cra Y47D, both compared to cells producing wild-type Cra, suggests that only part of the cellular Cra pool is phosphorylated, thereby potentially targeting parts of the Cra regulon to fine-tune expression rather than generating an all-or-none regulatory response. Indeed, we did not observe significant DE of several well-characterized Cra-controlled genes involved in the tricarboxylic acid (TCA) cycle, glycolysis, and gluconeogenesis that are known to be positively (*aceA*, *acnA*, *fbp*, *icdA*, *pckA*, *ppsA*, and *cydB*) and negatively (*acnB*, *eda*, *edd*, *eno*, *gapA*, *pfkA*, *ptsH*, and *pykF*) regulated by Cra (30).

10.1128/mBio.00097-18.8 TABLE S3  Comparison of DE genes in EHEC expressing Cra Y47F and Cra Y47D compared to EHEC expressing wild-type Cra (Venn diagram data). Download TABLE S3, XLSX file, 0.2 MB. Copyright © 2018 Robertson et al. 2018 Robertson et al. This content is distributed under the terms of the Creative Commons Attribution 4.0 International license.

### Phosphotyrosine-mediated regulation by Cra affects EHEC virulence gene expression.

**FIG 3:**
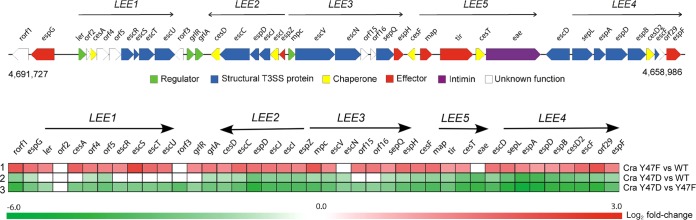
Cra phosphorylation negatively affects *LEE* expression. Diagram of the LEE PAI and heat map of *LEE* gene expression in EHEC producing nonphosphorylatable Cra Y47F versus wild-type Cra (row 1), phosphomimetic Cra Y47D versus wild-type Cra (row 2), and phosphomimetic Cra Y47D versus nonphosphorylatable Cra Y47F (row 3). LFCs are shown with red, green, and white indicating increased DE, decreased DE, and no significant difference in expression, respectively.

Among genes DE in response to regulation by Cra Y47 phosphorylation are genes related to virulence and various metabolic pathways (Fig. 2; Tables S2 and S3). The LEE PAI that encode the T3SS exhibited the greatest degree of differential expression, with all but 1 of the 41 *LEE* genes showing significantly lower expression in EHEC producing phosphomimetic Cra Y47D (Fig. 3). The finding that 32 of 41 *LEE* genes had significantly greater expression in cells producing nonphosphorylatable Cra Y47F than in cells producing wild-type Cra further supported a negative regulatory effect of Cra phosphorylation on *LEE* expression (Fig. 2A and 3; Table S4). These data are consistent with Cra Y47 affecting the production and secretion of *LEE*-encoded T3SS proteins (Fig. 1C). We also observed significant differential expression of the Ler-regulated prophage CP-933P gene *nleA*, which encodes the T3SS-secreted effector NleA (62, 63) (Table S4). Importantly, we detected significant differential expression of *ler*, encoding the activator Ler, which followed the expression pattern of *LEE* genes and *nleA* with reduced expression in cells producing phosphomimetic Cra Y47D and increased expression in cells producing nonphosphorylatable Cra Y47F (Fig. 3; Table S4). Given that the only identified Cra binding site within the LEE island is located upstream of the *ler* gene (*LEE1* operon) (31, 52), phosphotyrosine-mediated regulation by Cra likely controls the expression of T3SS genes by negatively modulating *ler* expression. Also, differential expression of *LEE* genes that encode the GrlA and GrlR transcriptional regulators (Fig. 3; Table S4), which fine-tune *ler* expression through a positive regulatory feedback loop (64), likely amplifies the impact of phosphotyrosine-mediated control of *ler* expression. Furthermore, the expression of chromosomal genes located on various prophages, including CP-933K, CP-933M, CP-933R, CP-933P, CP-933T, CP-933V, and CP-933X, was significantly decreased in cells producing phosphomimetic Cra Y47D, indicating that Cra phosphorylation affects the expression of prophage functions (Fig. 2A; Table S4).

10.1128/mBio.00097-18.9 TABLE S4  Selected genes that exhibit significant differential expression in cells producing wild-type Cra, Cra Y47F and Cra Y47D. Download TABLE S4, DOCX file, 0.02 MB. Copyright © 2018 Robertson et al. 2018 Robertson et al. This content is distributed under the terms of the Creative Commons Attribution 4.0 International license.

The global transcriptome data also revealed significant differential expression of Ler-activated T2SS genes *etpC* and *stcE* located on the pO157 virulence plasmid (11, 65, 66) (Fig. 2B; Table S4). The expression pattern of *stcE* and *etpC* mimics that of *LEE* with reduced transcript levels in EHEC producing phosphomimetic Cra Y47D and increased levels in EHEC producing nonphosphorylatable Cra Y47F. Given that no Cra DNA binding sites were identified in the regulatory region of *etpC* and *stcE* (52), phosphotyrosine-mediated regulation by Cra of *ler* expression also likely facilitates the observed regulatory effect on *stcE* and *etpC* expression. Indeed, LEE genes, along with *stcE* and *etpC*, comprised 95% of the Ler-regulated genes identified in a recent microarray analysis of the Ler regulon in EHEC (65), further indicating that phosphotyrosine-mediated regulation of these virulence genes by Cra occurs through Ler. We confirmed the differential expression of selected *LEE* genes, *nleA*, and *stcE* by quantitative reverse transcription-PCR (Fig. S2C). Also, pO157 genes involved in plasmid DNA replication initiation (*repA* and *repA4*) and various genes of unknown function (Z_L7074-Z_L7087) showed significantly decreased expression in EHEC producing phosphomimetic Cra Y47D, suggesting that phosphotyrosine-mediated regulation by Cra affects plasmid replication and other plasmid-encoded functions that have yet to be defined (Fig. 2B; Tables S2 and S3). Interestingly, the global transcriptome data revealed a regulatory effect of Cra phosphorylation on only two (*espG* and *ler*) of eight PAI genes previously reported as being directly regulated by Cra on the basis of a comparison of the transcriptomes of wild-type EHEC and a *cra* deletion mutant (52). Thus, only part of the Cra regulon responds to Cra tyrosine phosphorylation, further supporting the idea that phosphotyrosine-mediated regulation serves to fine-tune gene expression. Overall, phosphorylation of Cra Y47 affects the expression of at least 43 EHEC PAI genes that encode the T2SS and T3SS, which are essential for EHEC to achieve its fullest virulence potential.

### Phosphomimetic Cra negatively affects A/E lesion formation by EHEC.

**FIG 4:**
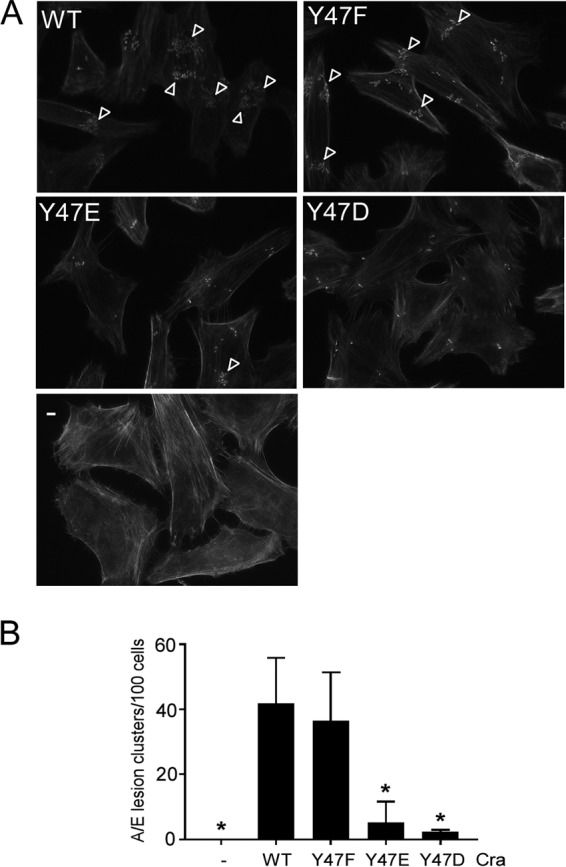
Phosphomimetic Cra negatively affects A/E lesion formation by EHEC. (A) A/E lesion formation on HeLa cell monolayers of EHEC producing no Cra (−), wild-type Cra, nonphosphorylatable Cra (Y47F), and phosphomimetic Cra (Y47D and Y47E). A/E lesions are visualized as condensed fluorescein isothiocyanate-phalloidin-stained actin. Arrowheads indicate clusters of at least eight A/E lesions in representative images of HeLa cells (*n* = 300). (B) A/E lesion formation efficiencies as determined by the number of clusters containing at least eight lesions per 100 HeLa cells. The standard deviations are indicated. The unpaired *t* test was used to determine statistically significant differences relative to EHEC expressing wild-type (WT) Cra with *P* < 0.05, as indicated by an asterisk.

The observed negative regulatory effect of Cra Y47 on *LEE* expression and T3SS protein production and secretion led us to determine whether phosphotyrosine-mediated regulation by Cra Y47 affects A/E lesion formation. We evaluated the abilities of strains producing wild-type Cra, nonphosphorylatable Cra (Y47F), and phosphomimetic Cra variants (Y47D and Y47E) to support A/E lesion formation by an EHEC *cra* mutant by using the fluorescent actin staining (FAS) assay (67, 68). EHEC strains producing phosphomimetic Cra Y47E and Y47D variants exhibited A/E lesion formation significantly decreased by 8- and 16-fold, respectively, relative to that of EHEC strains producing wild-type Cra (unpaired *t* test, *P* < 0.05) (Fig. 4), suggesting that Cra Y47 phosphorylation negatively affects A/E lesion formation. Nonphosphorylatable Cra Y47F did not significantly affect A/E lesion formation under these conditions (growth in Dulbecco’s modified Eagle’s medium [DMEM]). These data indicate that phosphotyrosine-mediated regulation by Cra affects the virulence potential of EHEC by reducing A/E lesion formation.

### Tyrosine phosphorylation diminishes Cra DNA binding.

**FIG 5:**
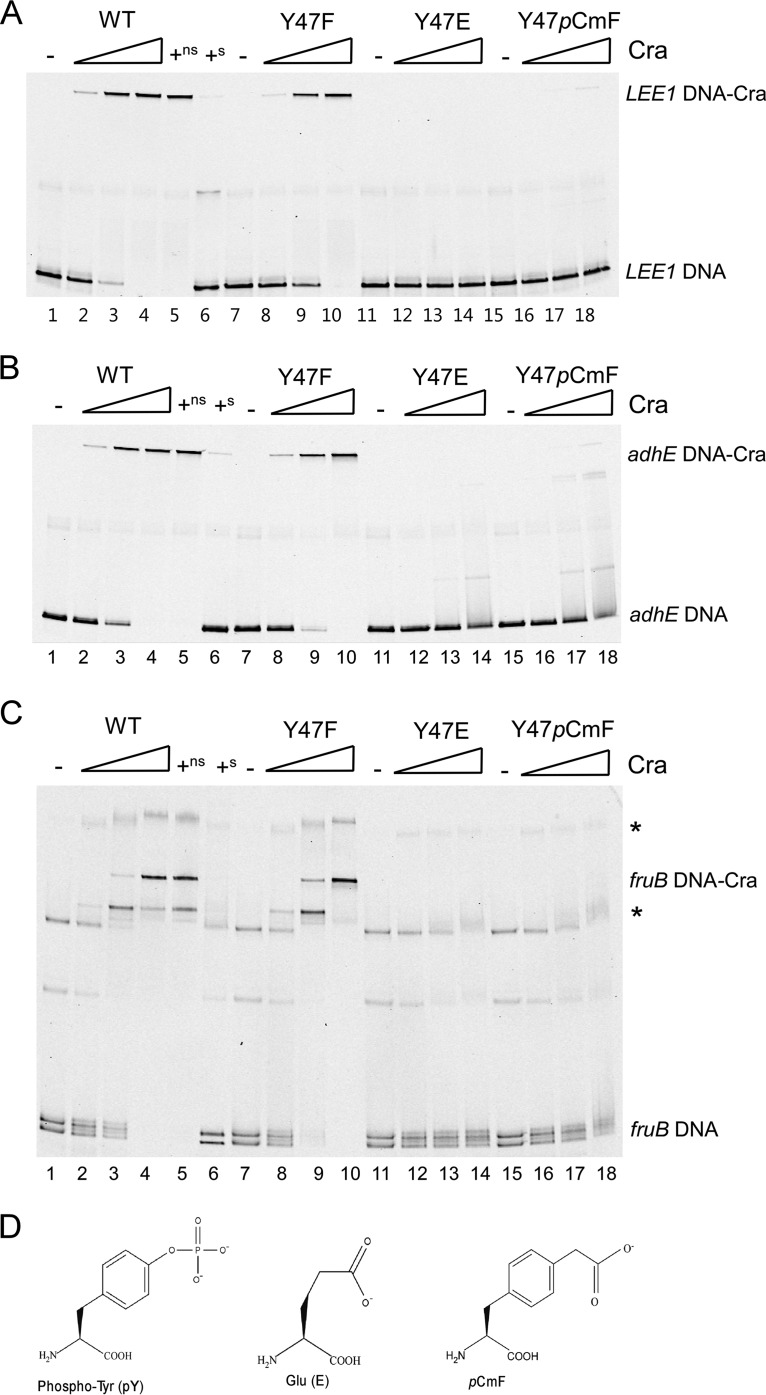
Tyrosine phosphorylation abolishes the DNA-binding capacity of Cra. The DNA-binding capacities of purified wild-type (WT) Cra and Y47-substituted Cra variants were evaluated by EMSAs with *LEE1* (A), *adhE* (B), and *fruB* (C) DNA targets. (D) Structures of phosphotyrosine, glutamate, and the phosphotyrosine analog *p*CmF used to substitute Cra Y47 to generate phosphomimetic Cra variants. Fluorescently labeled DNA was incubated with increasing concentrations (10, 75, and 200 nM) of wild-type Cra (lanes 2 to 6), Cra Y47F (lanes 8 to 10), Cra Y47E (lanes 12 to 14), and Cra Y47*p*CmF (lanes 16 to 18) and then subjected to gel electrophoresis. DNA binding specificity was determined by coincubating wild-type Cra (plus sign) with unlabeled nonspecific competitor DNA (ns; lane 5, *rssB*) and unlabeled specific competitor DNA (s; lane 6 *LEE1* [A], *adhE* [B], or *fruB* [C]). Locations of unbound and Cra-bound DNA are indicated. Asterisks designate protein-bound DNA subpopulations. The images shown are representative of at least three independent experiments.

Tyrosine residues in the LacI/GalR family regulators LacI and PurR that are equivalent to Cra Y47 have been demonstrated to interact with the DNA backbone (57, 69). We therefore determined the effect of Y47 phosphorylation on Cra DNA binding with electrophoretic mobility shift assays (EMSAs) by assessing the abilities of wild-type Cra and Y47 substitution variants to bind to fluorescently labeled target DNA fragments (70). Cra recognizes and binds to the 14-bp consensus sequence RSTGAAWCSNTHHW (71), which is present in the region upstream of *LEE1/ler* (31). The Cra binding site within the regulatory region of *LEE1* is of particular interest because phosphotyrosine-mediated regulation of *LEE1/ler* by Cra likely accounts for the observed differential expression of many Ler-regulated virulence genes (Fig. 2 to 3; Table S4). A search for genes with already known Cra DNA binding sites (48, 49, 52) among the DE genes identified in the present study only revealed a binding site in the regulatory region of *prpB*, which encodes a methylisocitrate lyase (48). However, we were unable to detect binding of Cra to *prpB* target DNA (Fig. S4A). In addition to the *LEE1* target, we therefore included two well-characterized Cra targets contained in the regulatory regions of *adhE* and *fruB* (71, 72). EMSAs revealed that increasing concentrations of wild-type Cra bound to *LEE1*, *adhE*, and *fruB* target DNA as expected (Fig. 5A to C, lanes 2 to 4). We verified the DNA-binding specificity of Cra to *LEE1*, *adhE*, and *fruB* target DNA by using unlabeled nonspecific (*rssB*) and specific fragments as competitor DNA (Fig. 5A to C, lanes 5 to 6). Nonphosphorylatable Cra Y47F bound to the DNA targets despite lacking the hydroxyl group of Y47 expected to interact with DNA (Fig. 5A to C, lanes 8 to 10). To establish whether the DNA-binding ability of Cra Y47F differs from that of wild-type Cra, we determined the protein dissociation constants by using the *LEE1* DNA target as previously described (10, 73, 74). The dissociation constants measured for wild-type Cra (111 ± 17 nM) and Cra Y47F (130 ± 17 nM) were not significantly different (*P* = 0.15) (unpaired *t* test, *P* < 0.05). These data indicate that hydrogen bond formation between the hydroxyl group of Y47 and DNA is dispensable for Cra DNA binding.

To determine whether phosphorylation of Cra Y47 affects Cra DNA binding, we used phosphomimetic Cra Y47E, which was unable to bind DNA fragments containing the regulatory regions of *LEE1*, *adhE*, and *fruB* (Fig. 5A to C, lanes 12 to 14). These data indicate that the presence of a negatively charged residue at position 47 diminishes Cra DNA binding. Yet, given that aspartate and glutamate do not completely mimic a phosphotyrosine structurally (Fig. 5D), the observed inability of Cra Y47E to bind DNA could be due to steric hindrance rather than the presence of a phosphorylated residue. To address this possibility, we took advantage of an approach successfully used in eukaryotic protein phosphorylation studies that includes the nonhydrolyzable phosphotyrosine analogue *p*-carboxymethylphenylalanine (*p*CmF), which is structurally and electrostatically similar to a phosphotyrosine (Fig. 5D) (75, 76). To incorporate *p*CmF at Cra position 47, we replaced codon 47 with an amber stop codon (UAG) and then used an orthogonal aminoacyl-tRNA synthetase-tRNA pair (aaRS/tRNA_CUA_) encoded by pEVOL-pCmF to incorporate *p*CmF at UAG (76). To ensure specific incorporation of *p*CmF, we used the genomically recoded strain C321.ΔA.exp, which has UAG reassigned as a sense codon to optimize the incorporation of unnatural amino acids (77). EMSAs revealed that Cra Y47*p*CmF containing the phosphotyrosine analogue, like Cra Y47E, exhibits diminished binding to Cra target DNA (Fig. 5A to C, lanes 16 to 18). These data indicate that it is tyrosine phosphorylation, rather than merely the presence of a negatively charged residue, that interferes with the DNA-binding ability of Cra.

Providing that glycolytic metabolites modulate the ability of Cra to bind DNA (30), Cra Y47 phosphorylation could, in theory, affect DNA binding through metabolite-mediated control instead of representing an independent mechanism. To assess this, we compared the DNA binding of wild-type Cra and that of nonphosphorylatable Cra Y47F in the presence of the glycolytic metabolite FBP. The presence of FBP reduced the binding of both wild-type Cra and Cra Y47F to *LEE1* DNA by 33% (Fig. S3, compare lanes 2 and 3 and lanes 6 and 7), whereas glucose-6-phosphate, which served as negative control, did not affect Cra DNA binding (Fig. S3, lanes 4 and 8). These findings indicate that Cra Y47 phosphorylation is not a prerequisite for metabolite-mediated control of Cra DNA binding, suggesting that phosphotyrosine-mediated regulation and metabolite-mediated regulation represent two independent mechanisms to control the DNA-binding capacity of Cra.

10.1128/mBio.00097-18.4 FIG S3  Catabolite repression of Cra occurs independently of Cra Y47-mediated control of Cra DNA binding. EMSAs were performed to assess the abilities of wild-type (WT) Cra and nonphosphorylatable Cra Y47F to bind the *LEE1* target in the presence of the metabolite FBP and glucose-6-phosphate (G6P), which served as a negative control. Wild-type Cra and Cra Y47F (170 nM) were incubated with 190 µM FBP (lanes 3 to 4) or G6P (lanes 7 to 8) prior to incubation with the *LEE1* DNA fragment. The positions of PAGE-resolved protein-bound and unbound DNA fragments are indicated. The images shown are representative of at least three independent experiments. Download FIG S3, TIF file, 0.5 MB. Copyright © 2018 Robertson et al. 2018 Robertson et al. This content is distributed under the terms of the Creative Commons Attribution 4.0 International license.

### Tyrosine phosphorylation affects the DNA-binding capacity of other LacI/GalR family regulators.

**FIG 6:**
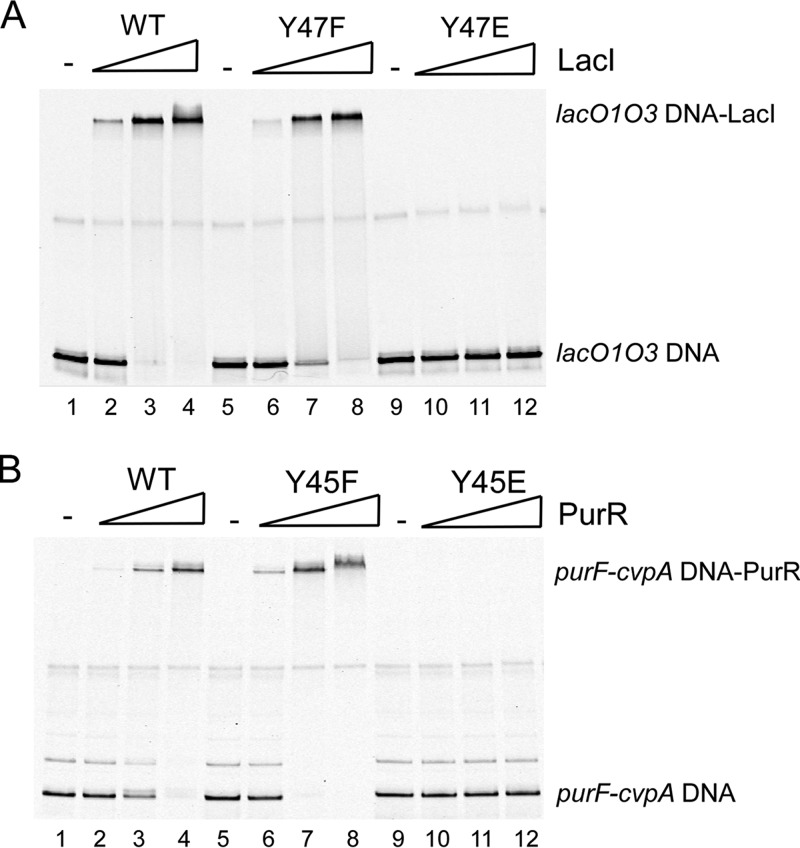
Phosphomimetic PurR and LacI negatively affect DNA binding. The abilities of wild-type (WT) and substituted variants of LacI Y47 (A) and PurR Y45 (B) to bind to, respectively, *lacO1O3* and *purF-cvpA* target DNA was determined by EMSAs. The purified wild-type proteins (LacI and PurR, lanes 2 to 4), nonphosphorylatable variants (LacI Y47F and PurR Y45F, lanes 6 to 8), and phosphomimetics variants (LacI Y47E and PurR Y45E, lanes 10 to 12) were bound to their respective target DNA at increasing concentrations (25, 150, and 300 nM). Positions of protein-bound and unbound DNA fragments are indicated. The images shown are representative of at least three independent experiments.

We previously also demonstrated phosphorylation of the PurR Y45 residue that is structurally equivalent to Cra Y47 (27), suggesting that tyrosine phosphorylation also could modulate the DNA binding capacity of other LacI/GalR regulators. To address this, we substituted the corresponding tyrosine residues in PurR (Y45) and LacI (Y47) with nonphosphorylatable (F) and phosphomimetic (E) residues and assessed the DNA binding of these proteins by EMSA. Whereas wild-type and nonphosphorylatable LacI and PurR variants bound to DNA at increasing concentrations (Fig. 6A and B, lanes 2 to 8), the phosphomimetic variants did not bind DNA (Fig. 6A and B, lanes 10 to 12), suggesting that tyrosine phosphorylation negatively controls the DNA-binding abilities of LacI and PurR. Furthermore, PurR containing the phosphotyrosine homologue *p*CmF in place of Y47 did not bind DNA, further supporting the regulatory effect of a phosphotyrosine (Fig. S4B, lanes 14 to 16). These findings are consistent with the observed effect of Y47-mediated regulation by Cra (Fig. 5A to C). Thus, our data suggest that tyrosine phosphorylation could provide a general regulatory mechanism for DNA binding of the LacI/GalR family in addition to ligand-mediated control.

10.1128/mBio.00097-18.5 FIG S4  EMSAs to assess Cra binding to *prpB* DNA and PurR-*p*CmF binding to *purF-cvpA* DNA. (A) The capacity of purified wild-type (WT) and Y47 substitution Cra variants to bind *prpB* target DNA was evaluated by EMSA. Fluorescently labeled *prpB* DNA was incubated with increasing concentrations (10, 75, and 200 nM) of wild-type Cra (lanes 2 to 4), Cra Y47F (lanes 6 to 8), Cra Y47E (lanes 10 to 12), and Cra Y47*p*CmF (lanes 14 to 16) and then subjected to gel electrophoresis. (B) The abilities of wild-type PurR and Y45-substituted variants to bind to *pyrF-cvpA* target DNA was determined by EMSA. Purified wild-type PurR (lanes 2 to 4) and PurR Y45F (lanes 6 to 8) and phosphomimetic PurR Y45E (lanes 14 to 16) variants were bound to their respective target DNA at increasing concentrations (25, 150, and 300 nM). Positions of protein-bound and unbound DNA fragments are indicated. Download FIG S4, TIF file, 0.6 MB. Copyright © 2018 Robertson et al. 2018 Robertson et al. This content is distributed under the terms of the Creative Commons Attribution 4.0 International license.

## DISCUSSION

**FIG 7:**
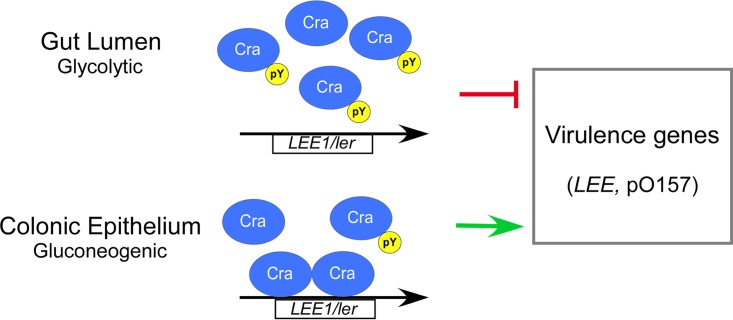
Model of phosphotyrosine-mediated regulation by Cra. Under glycolytic conditions typical of the environment of the colonic lumen where T3SS production is unnecessary, Cra Y47 phosphorylation diminishes Cra DNA binding to Cra targets, including *LEE1/ler*, and thereby provides a means in addition to catabolite repression to prevent the expression of virulence-associated genes, including those of the *LEE* and pO157. In the gluconeogenic environment of the colonization site Cra Y47 phosphorylation is likely reduced, which promotes the expression of virulence genes. Our model suggests that phosphotyrosine-mediated regulation represents a gradual rather than an all-or-none response to modulate Cra activity and, with that, fine-tune gene expression according to nutrient availability. The arrows and the T line indicate positive and negative regulation, respectively.

Successful host infection by pathogens relies on a highly integrated regulatory network that coordinates the expression of virulence and metabolic genes in response to nutrient-derived environmental cues. Specifically, EHEC links the expression of virulence factors to metabolic sensing through the regulator Cra by promoting the expression of virulence genes in response to the gluconeogenic environment at the colonization site, whereas glycolytic conditions prevent unnecessary expression in the colonic lumen (31). Here, we demonstrate that tyrosine phosphorylation negatively controls Cra DNA binding (Fig. 5), which is reflected in global differential gene expression in cells producing nonphosphorylatable Cra Y47F and phosphomimetic Cra Y47D (Fig. 2). Yet, our data indicate that only part of the Cra regulon, previously defined by using a *cra* deletion mutant (52), responds to phosphotyrosine-mediated regulation, suggesting that a fraction rather than the entire Cra pool is tyrosine phosphorylated. This notion is consistent with the findings of Soares et al., who reported a low median occupancy of Ser/Thr/Tyr phosphorylation sites in *E. coli* (<12%) (78). Given that Cra Y47 exhibits a regulatory effect in cells grown on glycolytic rather than gluconeogenic carbon sources (Fig. 1B), we speculate that a greater fraction of Cra protein is phosphorylated in response to glycolytic conditions, as illustrated in the proposed model (Fig. 7). Hence, changing Cra phosphorylation levels likely serves to fine-tune gene expression. Importantly, our data indicate that phosphorylation of Cra Y47 negatively affects the binding of Cra to the regulatory region of *LEE1/ler*, which encodes the major activator of LEE expression, Ler (Fig. 5A). Accordingly, phosphotyrosine-mediated regulation by Cra negatively affects *ler* expression and subsequently Ler-regulated genes that encode the T3SS, such as those on the LEE and *nleA* (Fig. 2A and 4). Hence, phosphotyrosine-mediated regulation by Cra negatively affects the production and secretion of T3SS proteins, resulting in decreased A/E lesion formation (Fig. 1 and 4). Given that untimely expression of the T3SS during infection likely provides a detrimental energy burden and may expose EHEC to the host immune system (26), Cra phosphorylation could provide an additional mechanism besides catabolite repression to prevent the activation of *LEE/ler* expression by Cra in a glycolytic environment typical of the colonic lumen. Moreover, Cra phosphorylation negatively affects the expression of Ler-regulated pO157 genes *stcE* and *etpC* (Fig. 2B), which are associated with the T2SS and involved in cell adherence and intimate attachment (79, 80). Thus, phosphotyrosine-mediated control by Cra is likely to affect the virulence potential of EHEC by fine-tuning the expression of genes that encode the T2SS and T3SS to prevent redundant expression in the glycolytic environment of the colonic lumen.

Apart from controlling the expression of virulence genes, our data suggest that Cra phosphorylation also likely affects the virulence potential of EHEC by indirectly affecting the expression of metabolic genes involved in the acquisition of gluconeogenic nutrients (Fig. 2A; Tables S2 and S3). In particular, phosphomimetic Cra negatively affects the expression of *eut* locus genes involved in ethanolamine transport and metabolism, which is known to provide a nitrogen source for EHEC under nutrient-limited conditions and thereby a competitive advantage over the commensal flora during infection (42). However, the link between Cra regulation and the *eut* operon remains unknown, as Cra is not known to control the expression of *eut* genes either directly or indirectly through regulation of *eutR*, which encodes a *eut* operon regulator (81). Furthermore, the global transcriptome data suggest that Cra tyrosine phosphorylation negatively affects the expression of *phn* operon genes involved in phosphonate metabolism, which supplies inorganic phosphate under phosphate-limited conditions (82, 83). Phosphomimetic Cra also negatively affects the expression of *prp* and *mhp* operon genes associated with the metabolism of the gluconeogenic nutrients propionate and phenylpropanoid compounds, which are degraded to TCA cycle substrates that can serve as an energy source under glycolytic nutrient deprivation (84, 85). Thus, phosphotyrosine-mediated regulation by Cra could also affect the virulence potential of EHEC by preventing the unnecessary expression of genes related to the transport and utilization of gluconeogenic nutrients under glycolytic conditions.

Phosphorylation of Cra Y47 positioned in the DNA-binding domain affects global transcription by interfering with the DNA-binding capacity of Cra (Fig. 5). This is consistent with data demonstrating that phosphorylation of a tyrosine residue located in the DNA-binding domain HTH motif of the *Bacillus subtilis* transcriptional regulator FatR disrupts DNA binding (86). Given that Cra Y47 is conserved in Gram-negative bacteria, phosphotyrosine-mediated control by Cra might also occur in other human pathogens such as *S. flexneri* and *Salmonella enterica* the virulence of which Cra influences (87–89). Furthermore, we showed that introduction of phosphomimetic residues at positions corresponding to Cra Y47 in the LacI/GalR family regulators PurR (Y45E) and LacI (Y47E) disrupts DNA binding, as observed for Cra (Fig. 6). These data suggest that tyrosine phosphorylation of LacI/GalR family regulators provides a regulatory mechanism to modulate DNA binding besides ligand-mediated allosteric control. PurR Y45 is phosphorylated under glycolytic conditions (27), where derepression of the PurR regulon to promote the expression of genes involved in purine synthesis is warranted. Although Cra Y47 was identified as phosphorylated under glycolytic conditions, other environmental cues such as oxygen availability might also induce Cra Y47 phosphorylation. Indeed, Carlson-Banning and Sperandio showed that aerobic conditions, representing the environment at the colonization site, are required for Cra-mediated activation of *LEE* expression, whereas anaerobic conditions mimicking those of the colonic lumen repress T3SS expression (53, 90, 91). Thus, oxygen sensing could provide a signal that favors Cra phosphorylation under anaerobic conditions to prevent the redundant T3SS expression in the colonic lumen. Indeed, tyrosine phosphorylation of the *Streptococcus pneumoniae* regulator CpsD, which is involved in capsular polysaccharide expression, is controlled by oxygen levels (92, 93). However, it is beyond the scope of this study to investigate the role of oxygen availability in Cra tyrosine phosphorylation. Also, future studies will define the tyrosine kinase(s) and phosphatase(s) that modulate the phosphorylation status of Cra. Overall, our data suggest that phosphotyrosine signaling provides an additional layer to the global regulatory network controlling virulence gene expression and thereby affects the virulence potential of EHEC. This notion is further supported by our previous finding that tyrosine phosphorylation of the global regulator SspA positively affects the production of *LEE*-encoded T3SS proteins and A/E lesion formation (27). In addition, it was recently demonstrated that tyrosine phosphorylation of the virulence gene regulator VirB and the T3SS ATPase Spa47 modulates the production and activity of the T3SS in *S. flexneri* (30), further emphasizing the importance of phosphotyrosine signaling in the control of enteric pathogen virulence.

In conclusion, we demonstrate that tyrosine phosphorylation of Cra negatively affects the expression of virulence-associated genes, including those that encode the T2SS and T3SS, to prevent redundant expression in glycolytic environments. Importantly, phosphotyrosine-mediated regulation affects A/E lesion formation and, with that, controls the virulence potential of EHEC. Specifically, tyrosine phosphorylation diminishes Cra DNA binding, suggesting that phosphotyrosine signaling could provide an additional mechanism to control the DNA-binding capacity of Cra besides catabolite repression. Our data suggest that Cra tyrosine phosphorylation fine-tunes gene expression in response to environmental cues such as glycolytic conditions to ensure optimal spatial-temporal expression of virulence-associated genes. Further studies are warranted to fully understand the extent and role of phosphotyrosine signaling in the regulation of EHEC virulence.

## MATERIALS AND METHODS

### Standard procedures.

Standard molecular biology techniques were used as previously described (94). Bacteria were grown at 37°C in LB medium, M9 medium, or DMEM (Corning catalog number 17207CU) supplemented with antibiotics (100 µg/ml ampicillin, 20 µg/ml chloramphenicol, and 30 µg/ml kanamycin) and carbon sources as needed. HeLa cells (ATCC CCL-2) were cultured in DMEM/F12 (Gibco catalog number 11330) supplemented with 10% fetal bovine serum (FBS), 100 U/ml penicillin, and 100 µg/ml streptomycin at 37°C in 7% CO_2_.

### Strain and plasmid construction.

The strains and plasmids used in this study (Table S5) were constructed by using standard genetic manipulations as described in Text S1. For biosafety, we used throughout this study an EHEC O157:H7 EDL933 strain with *stx*_1_ and *stx*_2_ deleted (TUV93-0) (61).

10.1128/mBio.00097-18.1 TEXT S1  Supplemental materials and methods used in this study. Download TEXT S1, DOCX file, 0.02 MB. Copyright © 2018 Robertson et al. 2018 Robertson et al. This content is distributed under the terms of the Creative Commons Attribution 4.0 International license.

10.1128/mBio.00097-18.10 TABLE S5  Strains, plasmids, and oligonucleotides used in this study. Download TABLE S5, DOCX file, 0.02 MB. Copyright © 2018 Robertson et al. 2018 Robertson et al. This content is distributed under the terms of the Creative Commons Attribution 4.0 International license.

### Western analysis.

Overnight cultures grown in LB were diluted 1:1,000 in M9 medium supplemented with a 0.4% carbon source (as indicated in the figure legends) and grown aerobically at 37°C to an optical density at 600 nm (OD_600_) of ~1. The total protein present in whole-cell lysates and culture supernatant fractions was precipitated with 5 and 10% (vol/vol) trichloric acid, respectively. Protein samples equivalent to 0.03 OD_600_ unit of culture were resolved on 4 to 20% Tris-HCl protein gels (Bio-Rad), and proteins were transferred onto an Immobilon-FL polyvinylidene difluoride membrane (Millipore). The membrane was blocked in Odyssey blocking buffer (LI-COR Biosciences); exposed to polyclonal antibodies specific to T3SS proteins (EspA, EspB, and Tir [95]), Cra, or GroEL (Sigma); and subsequently exposed to an Alexa Fluor 680-conjugated goat anti-rabbit secondary antibody (Invitrogen). A polyclonal rabbit antibody to purified Cra protein was generated by Lampire Biological Laboratories by using the EXPRESS-LINE service. GroEL served as an internal control for the total cellular protein loaded and was added to culture supernatants as a control for protein precipitation. Proteins were visualized and quantified with an Odyssey Infrared Imaging System and application software version 3.0 (LI-COR Biosciences) as recommended. Western analyses were carried out with four independent biological samples of each strain.

### RNA isolation and sequencing.

Overnight cultures of TUV93-0 Δ*cra*::FRT producing wild-type Cra (pAMH257), nonphosphorylatable Cra Y47F (pAMH258), or phosphomimetic Cra Y47D (pAMH268) were grown in LB and then diluted 1:100 in M9 with 0.4% glucose and grown aerobically at 37°C to an OD_600_ of ~1. Cells were treated with RNAlater (Ambion) in accordance with the manufacturer’s instructions. Total RNA was isolated from culture samples corresponding to ~2.3 × 10^10^ cells by a hot phenol extraction method as previously described (96). Contaminating DNA in the RNA preparations was removed with the Turbo DNA-free kit (Ambion). RNA samples were enriched by reducing rRNA levels with the Ribo-Zero magnetic kit for Gram-negative bacteria (Illumina). The DNA-free RNA samples were submitted for paired-end library construction with the TruSeq Library kit (Illumina) at the Institute for Genome Sciences Genomic Resource Center (http://www.igs.umaryland.edu/resources/grc/). The libraries were sequenced as 150-bp reads on the Illumina HiSeq 4000.

### RNA-Seq analyses.

The Illumina reads generated for each RNA sample were analyzed and compared by using an in-house Ergatis-based (97) RNA-Seq analysis pipeline as previously described (98). The reads were trimmed for quality with the FASTX-Toolkit (http://hannonlab.cshl.edu/fastx_toolkit/index.html). The reads were then aligned with the chromosome of EDL933 (AE005174.2) and plasmid pO157 (AF074613.1) with the Bowtie aligner (99). The number of reads that aligned with the protein-encoding regions was determined with HTSeq (100). The differential expression of each gene across the biological replicates was determined with DESeq (101). The LFCs were calculated for EHEC producing CraY47F versus wild-type Cra, CraY47D versus wild-type Cra, and CraY47D versus Cra Y47F. The genes were then filtered for further analysis to meet the following criteria: a minimum read count percentage of 0.1, transcript LFCs of ≥1 or ≤−1, *P* ≤ 0.01, and a false-discovery rate (FDR) of ≤0.05. Genes that met these criteria were identified as having significant differential expression. The circular displays of the significant LFCs for each gene of the EDL933 chromosome and the pO157 plasmid were generated with Circos 0.69-3 (102). Heat maps of the significant LFCs for the *LEE* genes were generated with MeV (103).

### FAS assay.

A/E lesions were detected with the FAS assay as previously described (67, 68). Briefly, overnight cultures of statically grown strains (~2 × 10^7^ bacteria, multiplicity of infection of ~10) were coincubated with HeLa cell monolayers in DMEM supplemented with 2% FBS. At 4 h postinfection, the monolayers were fixed in 4% formamide and F-actin was stained with Alexa Fluor 488 phalloidin (Invitrogen). The FAS assay was carried out independently at least three times for each strain. Samples were visualized with an Axioskop microscope equipped with a 40× objective, and images were captured with an AxioCam MR3 digital camera and AxioVision v 4.8 software (Carl Zeiss MicroImaging Inc.). Pedestal formation efficiency in each experiment was determined as the number of microcolonies containing at least eight lesions per 100 HeLa cells relative to the *cra* mutant producing wild-type Cra. The unpaired *t* test with a *P* value of <0.05 was used to determine statistical significance.

### Protein production and purification.

Recombinant wild-type Cra and Cra Y47 substitution variants were produced in TUV93-0 Δ*cra*::FRT from pAMH384 (Cra), pAMH385 (Cra Y47F), pAMH386 (Cra Y47E), and pAMH387 (Cra Y47D). Recombinant wild-type PurR and PurR Y45 substitution derivatives were produced in C321.ΔA.exp Δ*purR*::*kan* from pAMH413 (PurR), pAMH414 (PurR Y45F), and pAMH419 (PurR Y45E). Recombinant wild-type LacI and LacI Y47 substitution derivatives were produced in C321.ΔA.exp Δ*lacI*::*kan* from pAMH416 (LacI), pAMH417 (LacI Y47F), and pAMH420 (LacI Y47E). Overnight cultures were diluted 1:100 in LB containing ampicillin and grown aerobically at 37°C to an OD_600_ of ~0.8 prior to induction with 0.5 mM isopropyl-β-d-thiogalactopyranoside (IPTG) for 1 h. Cells were collected by centrifugation. To replace Cra Y47 with the phosphotyrosine analogue *p*CmF, we used an expression plasmid where the codon Y47 was replaced with an amber stop codon (Cra Y47TAG, pAMH390). To incorporate the phosphotyrosine analogue *p*CmF in place of Cra residue Y47, we used an orthogonal aminoacyl-tRNA synthetase--tRNA pair (aaRS/tRNA_CUA_) encoded by pEVOL-pCmF as previously described (75). We used strain C321.ΔA.exp (77) with *cra* deleted for the incorporation of *p*CmF (Table S4). Briefly, an overnight culture of C321.ΔA.exp Δ*cra*::*kan* (pAMH390, pEVOL-*p*CmF) was diluted 1:100 in LB containing ampicillin and chloramphenicol and grown aerobically in LB at 37°C to an OD_600_ of ~0.4. Then, 1 mM *p*CmF was added and the expression aaRS/tRNA_CUA_ was induced with 0.2% arabinose for 1 h prior to the induction of recombinant protein expression with 0.5 mM IPTG. Cells were grown for an additional 1 h and then harvested by centrifugation. The phosphotyrosine analogue *p*CmF was synthesized by AsisChem Inc. as previously described (75).

To purify recombinant proteins, cell pellets were suspended in buffer A (20 mM Tris HCl, 100 mM NaCl, 10% glycerol, 10 mM Na_4_P_2_O_7_, 1 mM Na_3_O_4_V, 0.5 mM PMSF, pH 8) containing 25 mM imidazole, cells were lysed by two passages through an LV1 microfluidizer (Microfluidics), and lysed cell suspensions were cleared by centrifugation at 26,500 × *g* for 15 min at 4°C. His-tagged proteins were purified with Ni-NTA agarose (Qiagen) in accordance with the manufacturer’s recommendations. Protein was eluted with buffer A containing 200 mM imidazole, resolved by SDS-PAGE with a 4 to 20% Tris-HCl precast gel (Bio-Rad), and visualized with GelCode Blue Stain Reagent (Thermo Scientific). Protein purity was about 95%, as estimated from stained gels. Purified protein was concentrated and buffer exchanged into buffer A with an Amicon Ultra Centrifugal Filter Device with a 30-kDa cutoff (Millipore) and then stored in buffer A containing 25% glycerol.

### EMSAs.

Fluorescently labeled oligonucleotides used for PCR amplification of DNA fragments containing Cra targets were prepared as previously described (104). The oligonucleotides used are listed in Table S5. Fragments containing Cra DNA-binding sites associated with the *LEE1* (−450 to −255) (31), *adhE* (−96 to +181) (72), and *fruB* (−43 to 234) (71) regulatory regions were PCR amplified from TUV93-0 genomic DNA (gDNA) with fluorescently labeled primer sets AH1384/AH1385, AH1382/AH1383, and AH1148/AH1149, respectively. Fragments containing the PurR and LacI DNA-binding sites located in the regulatory regions of *cvpA-purF* (−171 to 56) (105) and *lac* (−139 to 69) (106), respectively, were PCR amplified from TUV93-0 gDNA with fluorescently labeled primer sets AH1424/AH1425 and AH1426/AH1427. An unlabeled DNA fragment containing part of *rssB*, which served as a nonspecific DNA target, was amplified with oligonucleotides K5365/K5366. The DNA fragments were purified with G-50 spin columns (GE Healthcare). Purified wild-type and Y substitution derivatives of Cra, PurR, and LacI (the concentrations used are indicated in the figure legends) were incubated with 24 ng of fluorescently labeled target DNA fragment in binding buffer (60 mM HEPES-KOH, 25 mM MgCl_2_, 5 mM EDTA, 300 mM KCl, 50 µg/ml bovine serum albumin, 3 mM dithiothreitol, pH 7.5) for 20 min at room temperature. Unlabeled target DNA and nonspecific (*rssB*) DNA fragments added in 20-fold excess served as specific and nonspecific competitor DNA, respectively. DNA fragments were separated with a 4 to 20% TBE Criterion gel (Bio-Rad). Fluorescently labeled DNA fragments were visualized with an Odyssey Imaging System at 800 nm with application software version 3.0 (LI-COR Biosciences). EMSAs were carried out four times for each experiment with proteins from at least two different protein purification preparations.

### Accession number(s).

The Illumina reads obtained in this study have been deposited in the NCBI Short Read Archive under the accession numbers listed in Table S1. The RNA-Seq study is described under GEO accession number GSE103764.
