# Supplementary figures and images for: Phosphotyrosine-Mediated Regulation of Enterohemorrhagic Escherichia coli Virulence
Source: mBio. 2018 Feb 27;9(1):e00097-18. doi: 10.1128/mBio.00097-18 (PMC5829826; doi:10.1128/mBio.00097-18)

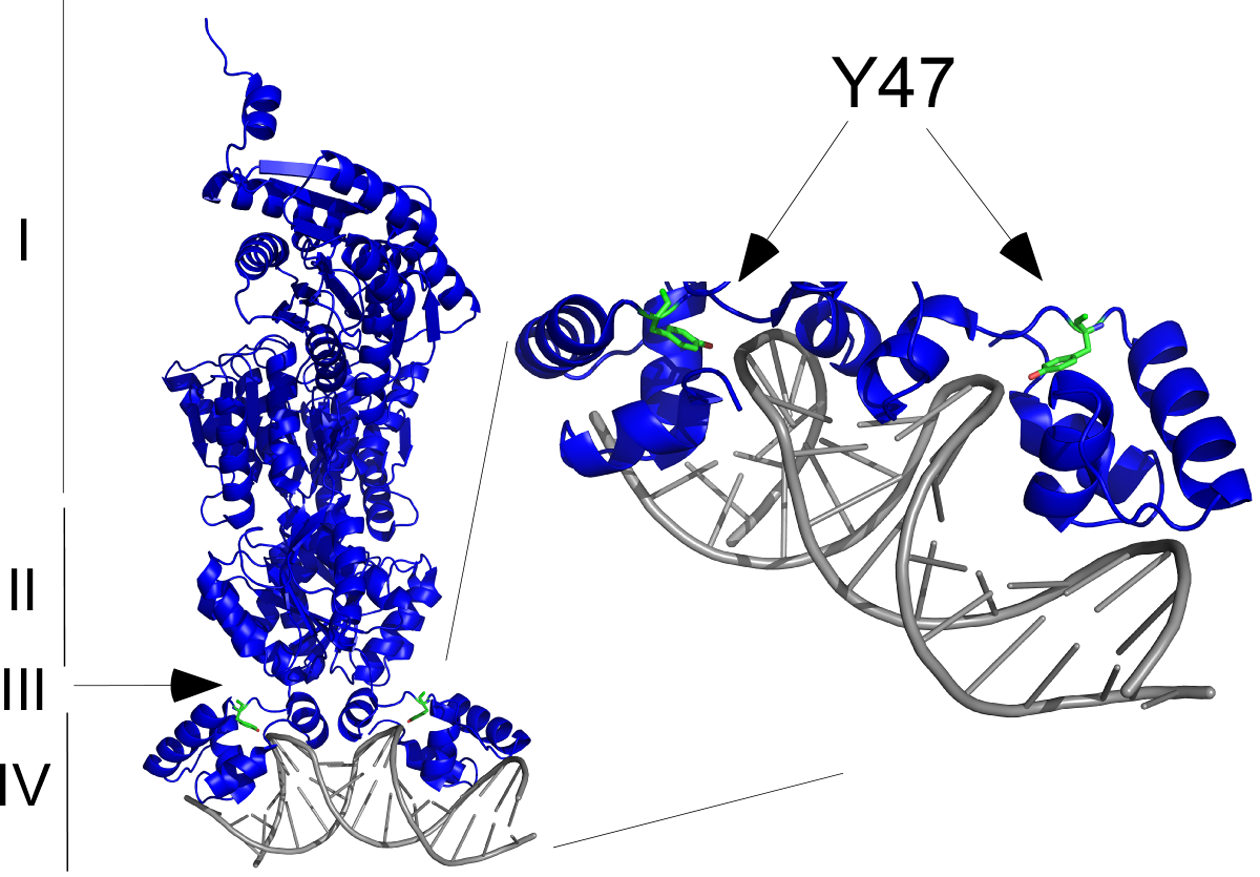

Supplement: FIG S1 [file mbo001183745sf1.tif]

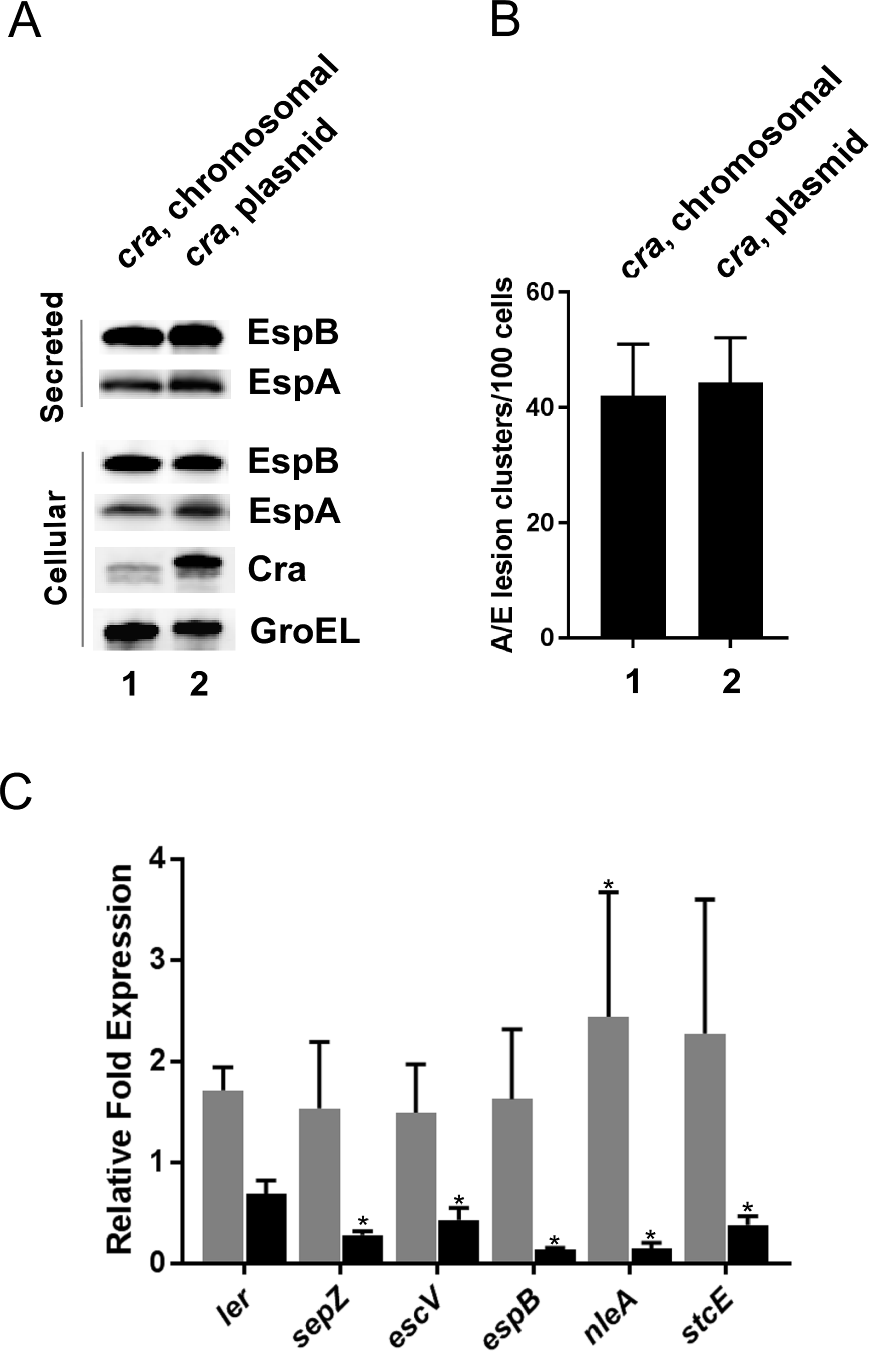

Supplement: FIG S2 [file mbo001183745sf2.tif]

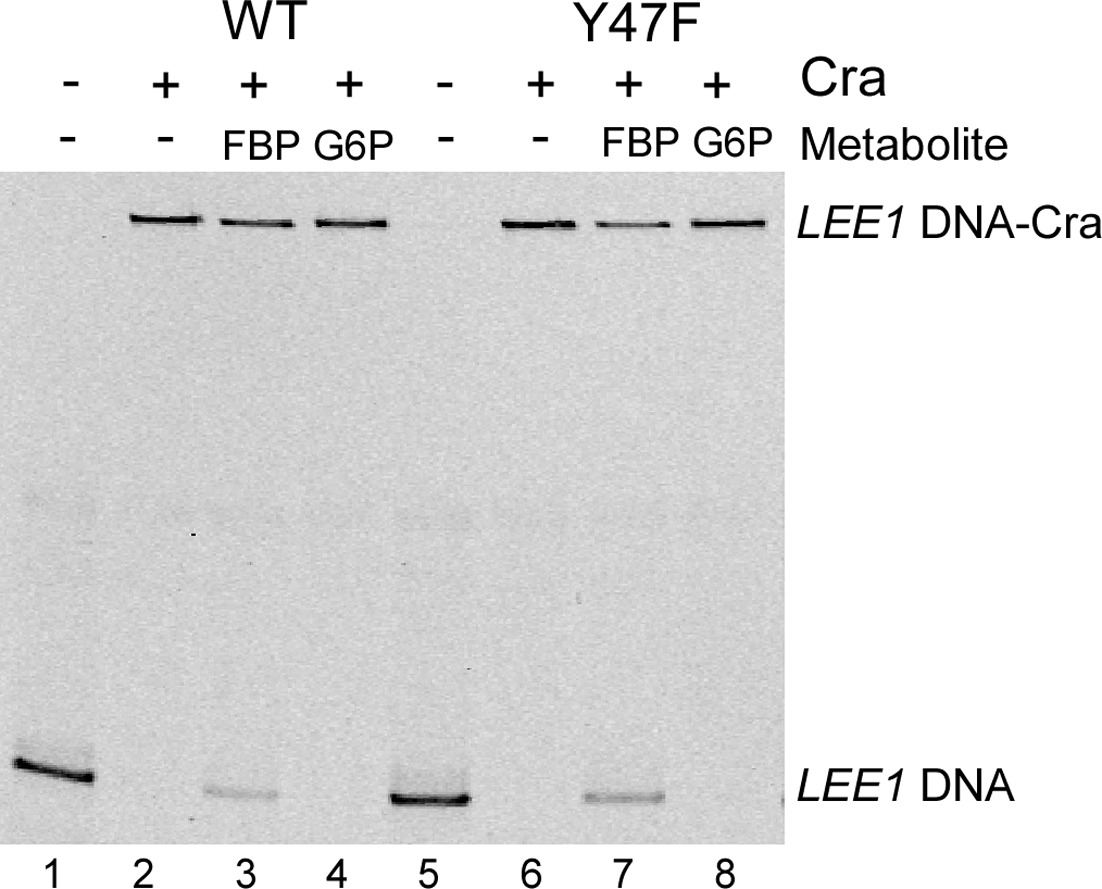

Supplement: FIG S3 [file mbo001183745sf3.tif]

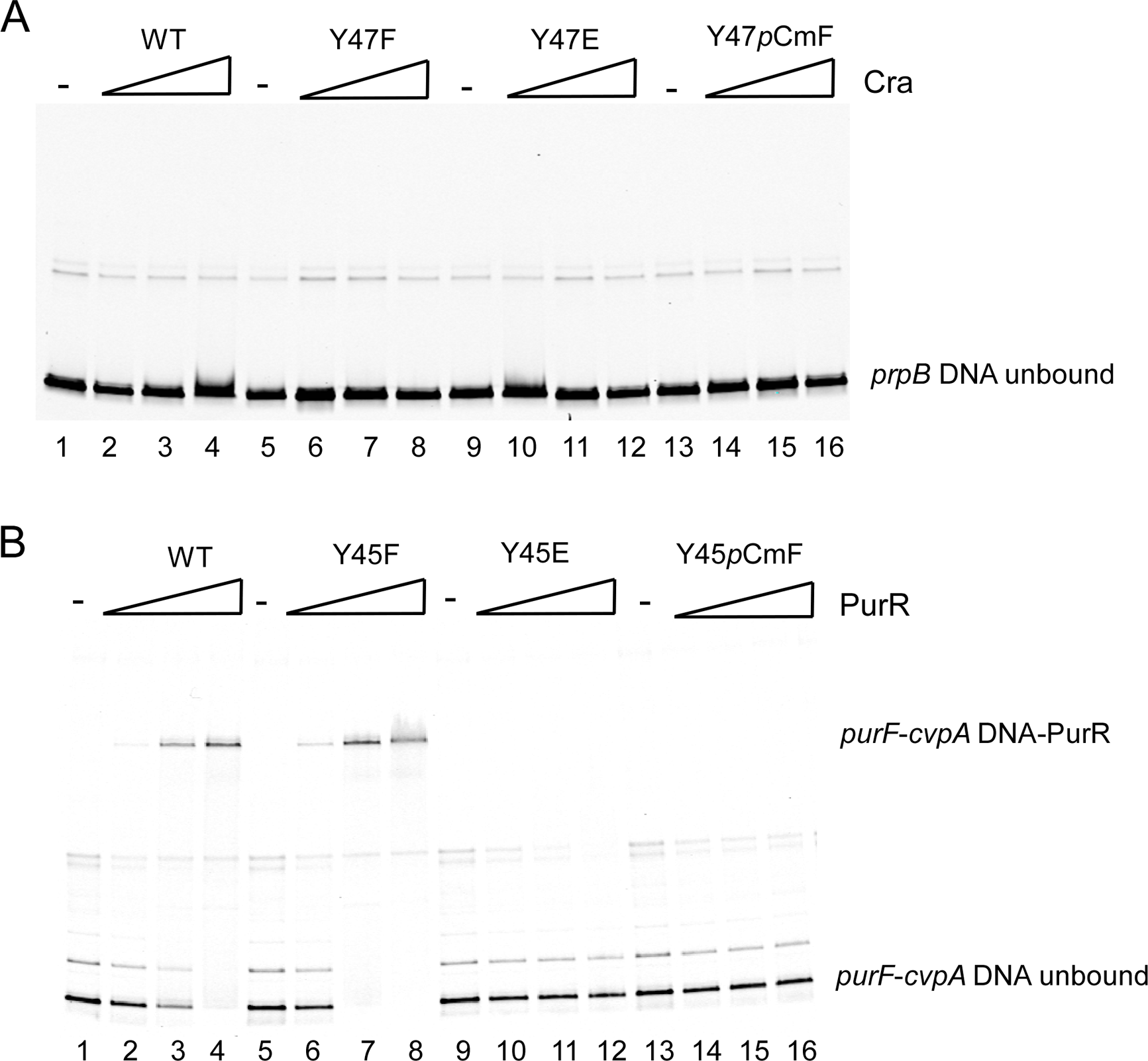

Supplement: FIG S4 [file mbo001183745sf4.tif]
