# Supplementary material for: Phosphotyrosine-Mediated Regulation of Enterohemorrhagic Escherichia coli Virulence
Source: mBio. 2018 Feb 27;9(1):e00097-18. doi: 10.1128/mBio.00097-18 (PMC5829826; doi:10.1128/mBio.00097-18)
Supplement: TABLE S4 [file mbo001183745st4.docx]

**Table S4.** Selected pathogenicity island and pO157 genes that exhibit significant differential expression in cells producing CraY47F and CraY47D.

| **Accession** | | **Gene** | **Protein function** | **Log_2_ fold-change^a^** | | |
| --- | --- | --- | --- | --- | --- | --- |
|  | | | | Cra Y47D vs Y47F | Cra Y47D vs WT | Cra Y47F vs WT |
| *LEE pathogenicity island* | | | |  |  |  |
| Z5143 | *rorf1* | | Unknown function | -4.24 | -2.23 | 2.03 |
| Z5142 | *espG* | | Type III secretion system secreted effector | -2.80 | -1.22 | 1.60 |
| Z5140 | *ler* | | Transcriptional regulator | -1.76 | NS | 1.30 |
| Z5139 | *orf2* | | Unknown function | NS | NS | NS |
| Z5138 | *cesA* | | Type III secretion system chaperone | -2.61 | NS | 1.85 |
| Z5137 | *orf4* | | Unknown function | -2.67 | -1.20 | 1.48 |
| Z5136 | *orf5* | | Unknown function | -3.08 | -1.71 | 1.39 |
| Z5135 | *escR* | | Type III secretion system inner membrane protein | -2.33 | NS | 1.29 |
| Z5134 | *escS* | | Type III secretion system inner membrane protein | -2.54 | NS | 2.35 |
| Z5133 | *escT* | | Type III secretion system inner membrane protein | -2.54 | NS | 1.75 |
| Z5132 | *escU* | | Type III secretion system inner membrane protein | -2.65 | NS | 1.54 |
| Z5131 | *rorf3* | | Unknown function | -3.39 | NS | NS |
| Z5129 | *grlR* | | Transcriptional regulator | -2.08 | NS | 1.24 |
| Z5128 | *grlA* | | Transcriptional regulator | -2.89 | -1.42 | 1.49 |
| Z5127 | *cesD* | | Type III secretion system chaperone | -2.54 | -1.32 | 1.24 |
| Z5126 | *escC* | | Type III secretion system outer membrane ring protein | -3.26 | -1.56 | 1.72 |
| Z5125 | *sepD* | | Type III secretion system secretion switching protein | -4.54 | -2.95 | 1.61 |
| Z5124 | *escJ* | | Type III secretion system bridge between inner and outer membrane | -3.73 | -2.21 | 1.54 |
| Z5123 | *escI* | | Type III secretion system inner rod protein | -3.68 | -2.24 | 1.46 |
| Z5122 | *sepZ* | | Type III secretion system secreted effector | -3.65 | -2.32 | 1.34 |
| Z5121 | *mpc* | | Type III secretion system regulator | -3.64 | -1.63 | 2.02 |
| Z5120 | *escV* | | Type III secretion system inner membrane channel protein | -3.17 | -1.91 | 1.28 |
| Z5119 | *escN* | | Type III secretion system ATPase | -2.24 | -1.47 | NS |
| Z5118 | *orf15* | | Unknown function | -2.34 | NS | 1.48 |
| Z5117 | *orf16* | | Unknown function | -2.18 | NS | 1.52 |
| Z5116 | *sepQ* | | Type III secretion system component | -2.47 | -1.32 | 1.16 |
| Z5115 | *espH* | | Type III secretion system secreted effector | -3.18 | -1.53 | 1.66 |
| Z5114 | *cesF* | | Type III secretion system chaperone | -3.50 | -1.98 | 1.53 |
| Z5113 | *map* | | Type III secretion system secreted effector | -3.71 | -2.08 | 1.64 |
| Z5112 | *tir* | | Intimin receptor | -2.93 | -1.86 | 1.09 |
| Z5111 | *cesT* | | Type III secretion system chaperone | -4.04 | -2.87 | 1.18 |
| Z5110 | *eae* | | Intimin adhesin | -4.23 | -3.35 | NS |
| Z5109 | *escD* | | Type III secretion system inner membrane ring protein | -3.62 | -2.09 | 1.55 |
| Z5108 | *sepL* | | Unknown function | -4.64 | -3.10 | 1.56 |
| Z5107 | *espA* | | Type III secretion system needle filament protein | -5.34 | -4.05 | 1.30 |
| Z5106 | *espD* | | Type III secretion system pore protein | -4.92 | -3.90 | 1.03 |
| Z5105 | *espB* | | Type III secretion system pore protein | -4.27 | -3.17 | 1.11 |
| Z5104 | *cesD2* | | Unknown function | -4.22 | -2.94 | 1.30 |
| Z5103 | *escF* | | Type III secretion system effector protein | -4.18 | -2.51 | 1.68 |
| Z5102 | *orf29* | | Unknown function | -4.42 | -2.35 | 2.09 |
| Z5100 | *espF* | | Type III secretion system secreted effector | -3.17 | -1.82 | 1.37 |
| *Non-LEE pathogenicity island* | | | |  |  |  |
| Z6024 | *nleA* | | Type III secretion system effector encoded by prophage CP-933P | -5.89 | -3.96 | 1.94 |
| Z0951 | *Z0951* | | Putative exonuclease encoded by prophage CP-933K | NS | -2.77 | NS |
| Z0979 | *Z0979* | | Putative tail component of prophage CP-933K | NS | -2.04 | NS |
| Z1383 | *Z1383* | | Unknown protein encoded by cryptic prophage CP-933M | -1.35 | -1.34 | NS |
| Z1891 | *Z1891* | | Putative tail component of prophage CP-933X | NS | -1.80 | NS |
| Z2354 | *Z2354* | | Putative partial tail component of prophage CP-933R | -2.00 | -2.88 | NS |
| Z2361 | *Z2361* | | Putative capsid assembly protein of prophage CP-933R | -1.30 | -1.85 | NS |
| *Plasmid pO157* | | |  |  |  |  |
| Z_L7031 | *stcE* | | Type II secretion system secreted effector | -2.43 | -1.38 | 1.07 |
| Z_L7032 | *etpC* | | Type II secretion system protein | -1.70 | NS | 1.15 |
| Z_L7007 | *repA* | | Plasmid replication initiation protein | -3.03 | -3.40 | NS |

^a^NS indicates that value for differential expression was not significant
