# Supplementary material for: Phosphotyrosine-Mediated Regulation of Enterohemorrhagic Escherichia coli Virulence
Source: mBio. 2018 Feb 27;9(1):e00097-18. doi: 10.1128/mBio.00097-18 (PMC5829826; doi:10.1128/mBio.00097-18)
Supplement: TEXT S1 [file mbo001183745s1.docx]

Supplemental Material and Methods

**Strain and plasmid constructions.**

Oligonucleotides, bacterial strains and plasmids used in this study are listed in Table S5.

*Strain constructions.* Nonpolar deletion mutant derivatives of *cra, purR* and *lacI* were constructed by Lambda Red-mediated recombination in TUV93-0 and/or C321.∆A.exp as described (1). TUV93-0 is a derivative of EHEC O157:H7 strain EDL933 that is deleted for *stx1* and *stx2* (2), and C321.∆A.exp is a genomically recoded *E. coli* MG1655 derivative with all UAG codons reassigned as sense (3). Briefly, DNA fragments encoding a kanamycin resistance cassette flanked by Flipase Recognition Target (FRT) site-inverted repeats and sequences homologous to regions flanking *cra, purR* and *lacI*, were PCR-amplified with from pKD13 using primer sets AH1128/AH1129, AH1404/AH1405 and AH1414/AH1415 respectively. The DNA fragments were electroporated into the target strains, where the Red recombinase system was expressed from pKD46, and recombinants were selected and purified on L-agar plates containing 30 µg/ml kanamycin. Gene deletions of *cra, purR* and *lacI* were verified by PCR amplification analysis using primer sets AH1131/AH1175, AH1406/AH1407 and AH1416/AH1417, respectively. The kanamycin resistance marker was eliminated from TUV93-0 Δ*cra*::*kan*, TUV93-0 using a pCP20-encoded FLP flipase (1) generating the kanamycin sensitive derivative TUV93-0 *cra*::FRT.

*Plasmid constructions.* Complementation plasmids expressing wild type and mutant *cra* from the native promoter were constructed using the low-copy number vector pSec10 (4). A 1.3 kb fragment encoding wild type *cra-his* and 284 bp of the upstream regulatory region was PCR amplified from TUV93-0 gDNA using oligos AH1157/AH1175, digested with *Sal*I and *Hind*III, and cloned into the corresponding sites of pSec10 (pAMH257). Fragments encoding Cra Y47 substitutions were generated by a two-step PCR process. The first fragment for each construct encoding Y47F, Y47D and Y47E substitutions were PCR amplified using primer sets AH1175/AH1133, AH1175/AH1239 and AH1175/AH1240 respectively, and digested with *Pvu*II and *Sal*I. The second fragment encoding the region downstream of Y47 was PCR amplified with AH1132/AH1157, and digested with *Pvu*II and *Hind*III. The two fragments were then ligated using T4 DNA ligase to generate full length *cra*. Fragments encoding the Cra substitutions were then PCR amplified with AH1157/AH1175 using the ligated fragments as template, digested with *Sal*I and *Hind*III, and cloned into the corresponding sites of pSec10 to generate pAMH258 (*cra* Y47F), pAMH267 (*cra* Y47D) and pAMH268 (*cra* Y47E). Wild type Cra and the Y47-substituted derivatives were produced from low-copy number plasmids at levels similar to that of Cra expressed from the chromosome (data not shown).

Expression plasmids used for the production of N-terminally His-tagged wild type and Y47-substituted Cra derivatives were constructed using pAMH383, a derivative of pQE80 (Qiagen) with the region encoding the C-terminal His-tag deleted. Expression vector pAMH383 was generated by reverse PCR amplification of pQE80 using primers AH1379 and AH1380, the fragment was phosphorylated and ligated. Expression plasmids producing N-terminally His-tagged Cra (pAMH384), Cra Y47F (pAMH385), Cra Y47E (pAMH386) and Cra Y47E (pAMH387) were generated by PCR amplifying DNA fragments from respectively pAMH257, pAMH258, pAMH268 and pAMH267 using primer set AH1134/AH1157, digesting the fragments with *BamH*I and *Hind*III, and cloning into the corresponding sites of pAMH383. Plasmid pAMH390 (Cra Y47TAG) was generated by PCR amplification of two sub-fragments from TUV93-0 gDNA using primer sets AH1134/AH1376 and AH1132/AH1157, digestion of the fragments with *Bam*HI/*Pvu*II and *Hind*HIII/*Pvu*II respectively, and ligation of the two fragments. The fragment encoding Cra Y47TAG was then PCR amplified with AH1134/AH1157 from the ligated fragments, digested with *BamH*I/*Hind*III and cloned into pAMH383. Expression plasmids encoding N-terminally His-tagged wild type PurR (pAMH413) and LacI (pAMH416) were constructed by PCR amplification of fragments encoding the respective genes from TUV93-0 gDNA using oligos AH1408/AH1409 and AH1418/AH1419, digestion of the fragments with *BamH*I/*Hind*III followed by cloning into pAMH383. Plasmids encoding Y45-substituted PurR derivatives were generated by a two-step PCR process. The first fragment for each construct encoding PurR Y45F and Y45E was PCR amplified using primer sets AH1408/AH1411 and AH1408/AH11413, respectively. The second fragment encoding the region downstream of Y47 was PCR amplified with AH1132/AH1157, and phosphorylated with T4 kinase. The two fragments were then ligated using T4 DNA ligase to generate full length *purR* derivatives. Fragments encoding the PurR Y45 substitutions were then PCR amplified with AH1408/AH1409 from the ligations, digested with *Bam*HI/*Hind*III, and cloned into pAMH383 to generate pAMH414 (*purR* Y45F) and pAMH419 (*purR* Y45E). Plasmids encoding Y47-substituted LacI derivatives were also generated by the two-step PCR process. The first fragment for each construct encoding LacI Y47F and Y47E were PCR amplified using primer sets AH1418/AH1421 and AH1418/AH11423, respectively. The second fragment encoding the region downstream of LacI Y47 was PCR amplified with AH1419/AH1420, and phosphorylated with T4 kinase. The two fragments were then ligated using T4 DNA ligase to generate full length *lacI* derivatives. Fragments encoding the LacI Y47 substitutions were then PCR amplified with AH1418/AH1419 from the ligations, digested with *Bam*HI/*Hind*III, and cloned into pAMH383 to generate pAMH417 (*lacI* Y47F) and pAMH420 (*lacI* Y47E). Constructs were verified by DNA sequencing.

**Quantitative Real-Time reverse transcription-PCR analyses.**

Quantitative Real-Time reverse transcription-PCR (qRT-PCR) was performed in a one-step reaction using the Brilliant II SYBR Green QRT-PCR Master Mix 1-Step Kit (Agilent) and a Chromo 4^TM^ real-time detector attached to a PTC-200 DNA engine base (MJ Research/BioRad) according to manufactures’ recommendation. The qRT-PCR reactions were carried out in technical duplicates on RNA isolated from at least three independent cultures for each construct. Transcripts encoding *ler, sepZ, escV, espB, nleA, stcE* and *rpoB* were detected using the primer pairs K5359/K5360, K5833/K5834, K5831/K5832, K5361/K5362, AH1475/AH1476, AH1495/AH1496 and K6088/K6089, respectively (Table S5). Template specificity for the pairs was verified by melting curve analysis. Data were collected using the MJ Opticon Monitor analysis software version 3.1 (BioRad), normalized to transcript levels of *rpoB*, and analyzed using the comparative critical cycle threshold (*C*_T_) method (5). The expression levels of target genes in wild type and mutant derivatives were compared using the relative quantification method. Statistical significance between means was calculated using the unpaired *t*-test with a threshold *P* value of < 0.05. Relative expression levels are expressed as the means ± the standard deviation of the ΔΔ*C*_T_  value.

**References**

1. **Datsenko, KA and Wanner, B. L.** 2000. One-step inactivation of chromosomal genes in *Escherichia coli* K-12 using PCR products. Proc.Natl.Acad.Sci.U.S.A **97**:6640-6645.

2. **Donohue-Rolfe, A, Kondova, I., Oswald, S., Hutto, D., and Tzipori, S.** 2000. *Escherichia coli* O157:H7 strains that express Shiga toxin (Stx) 2 alone are more neurotropic for gnotobiotic piglets than are isotypes producing only Stx1 or both Stx1 and Stx2. J.Infect.Dis. **181**:1825-1829.

3. **Lajoie, MJ, Rovner, A. J., Goodman, D. B., Aerni, H. R., Haimovich, A. D., Kuznetsov, G., Mercer, J. A., Wang, H. H., Carr, P. A., Mosberg, J. A., Rohland, N., Schultz, P. G., Jacobson, J. M., Rinehart, J., Church, G. M., and Isaacs, F. J.** 2013. Genomically recoded organisms expand biological functions. Science **342**:357-360.

4. **Stokes, MG, Titball, R. W., Neeson, B. N., Galen, J. E., Walker, N. J., Stagg, A. J., Jenner, D. C., Thwaite, J. E., Nataro, J. P., Baillie, L. W., and Atkins, H. S.** 2007. Oral administration of a *Salmonella enterica*-based vaccine expressing *Bacillus anthracis* protective antigen confers protection against aerosolized *B. anthracis*. Infect.Immun. **75**:1827-1834.

5. **Livak, KJ and Schmittgen, T. D.** 2001. Analysis of relative gene expression data using real-time quantitative PCR and the 2(-Delta Delta C(T)) Method. Methods **25**:402-408.
