# Supplementary material for: Phosphotyrosine-Mediated Regulation of Enterohemorrhagic Escherichia coli Virulence
Source: mBio. 2018 Feb 27;9(1):e00097-18. doi: 10.1128/mBio.00097-18 (PMC5829826; doi:10.1128/mBio.00097-18)
Supplement: TABLE S5 [file mbo001183745st5.docx]

**Table S5.** Strains, plasmids and oligonucleotides used in this study.

| **Strain or plasmid** | | **Relevant characteristic(s)** | **Reference or source** | |
| --- | --- | --- | --- | --- |
|  | |  |  | |
| Strains: | |  |  | |
| TUV93-0 | | EHEC O157:H7 strain EDL933Δ*stx1* Δ*stx2* | (1) | |
| C321∆exp | | Recoded *E. coli* MG1655 with all UAG codons and RF1 removed. | (2) | |
| AMH187 | | TUV93-0 Δ*cra::kan* | This study | |
| AMH188 | | TUV93-0 Δ*cra::*FRT | This study | |
| AMH205 | | C321.∆A.exp Δ*cra::kan* | This study | |
| AMH206 | | C321.∆A.exp Δ*purR::kan* | This study | |
| AMH207 | | C321.∆A.exp Δ*lacI::kan* | This study | |
|  | |  |  | |
| Plasmids: | |  |  | |
| pCP20 | | FLP-flipase expressing plasmid; *repA101* (ts) P*_λR_ -FLP* *λ* *c*I857 | (3) | |
| pEVOL-*p*CmF | | Plasmid expressing the orthogonal aminoacyl-tRNA synthethase-tRNA pair aaRS/tRNA_CUA_. Used for incorporation of *p*CmF at UAG. | (4) | |
| pKD13 | | FRT-flanked *kan* resistance cassette | (5) | |
| pKD46 | | λ Red recombinase expressing plasmid; *repA101* (ts) P*_araB_-gam-bet-exo* | (5) | |
| pQE80 | | Expression vecor P*_tac_*, *lacI^q^* | Qiagen | |
| pSec10 | | Low copy number plasmid, pSC10*ori* | (6) | |
| pSec10* | | pSec10 derivative with parts of *clyA* deleted. Used as vector control | (7) | |
| pAMH257 | | pSec10::*cra* | This study | |
| pAMH258 | | pSec10::*cra*Y47F | This study | |
| pAMH267 | | pSec10::*cra*Y47D | This study | |
| pAMH268 | | pSec10::*cra*Y47E | This study | |
| pAMH383 | | pQE80 deleted for the sequence encoding the the N-terminal His-tag | This study | |
| pAMH384 | | pAMH383::*cra* | This study | |
| pAMH385 | | pAMH383::*cra*Y47F | This study | |
| pAMH386 | | pAMH383::*cra*Y47E | This study | |
| pAMH387 | | pAMH383::*cra*Y47D | This study | |
| pAMH390 | | pAMH383::*cra*Y47TAG | This study | |
| pAMH413 | | pAMH383::*purR* | This study | |
| pAMH414 | | pAMH383::*purR*Y45F | This study | |
| pAMH416 | | pAMH383::*lacI* | This study | |
| pAMH417 | | pAMH383::*lacI*Y47F | This study | |
| pAMH419 | | pAMH383::*purR*Y45E | This study | |
| pAMH420 | | pAMH383::*lacI*Y47E | This study | |
| **Name** | **Oligonucleotide sequence (5’ to 3’)** | | |  |
|  |  | | |  |
| AH1128 | CCAGTACAATGGCTATGGTTTTTACATTTTACGCAAGGGGCAATTGTGT  CCGGGGATCCGTCGACCT | | |  |
| AH1129 | CGCCAGAGTGAAATTCACCTGGCGCGTATTTTTGTTCGCAGCTTAGTGTA  GGCTGGAGCTGCTTCG | | |  |
| AH1132 | GTGGCAGCTGGGCTTCGTGCT | | |  |
| AH1131 | CACCGAAGCTTGATGTCCAGTCCCGTACTCTAC | | |  |
| AH1133 | AGCCCAGCTGCCACGGCGTTCGGGTGCTAATTGT | | |  |
| AH1134 | AGCAGGATCCATGAAACTGGATGAAATCGCTCGGCT | | |  |
| AH1148 | TTTACGGCTTTCCTTGCGTGC | | |  |
| AH1149 | CAGCATGCCATTGACGTAGCC | | |  |
| AH1157 | CAGTAAGCTTAGTGATGGTGATGGTGATGGCTACGGCTGAGCACACCG | | |  |
| AH1175 | CATGTGTCGACGATGCATTTTTAGCATCGATTCGCGATGT | | |  |
| AH1239 | AGCCCAGCTGCCACGGCGTTCGGGTGGTCATTGTG | | |  |
| AH1240 | AGCCCAGCTGCCACGGCGTTCGGGTGCTCATTGTG | | |  |
| AH1376 | AGCCCAGCTGCCACGGCGTTCGGGTGCTAATTGTGCT | | |  |
| AH1382 | CACCTCCTAACTACTTAAAATTGCTATCATTCGT | | |  |
| AH1383 | GCGCACACACTCACTGTGATTTACTA | | |  |
| AH1384 | CATGTGCTGCGACTGCGTTCG | | |  |
| AH1385 | GCATTTCCATTTAGTTAAGCGTTTCTCTTTATAAGATAGATCTC | | |  |
| AH1404 | TTTGCAGGAGCTGAAGTTAGGGTCTGGAGTGAAATGGAATGTCCGGGG  ATCCGTCGACCT | | |  |
| AH1405 | GCCGGAAGAGACTCCCGCAACGGGTGATTAACGACGATAGTGTAGGCT  GGAGCTGCTTCG | | |  |
| AH1406 | CAGTGTCGACCGTCATTACGCGATATTCATTAAAGTGG | | |  |
| AH1407 | CAGGAAGCTTCGATAGTGAGACGCTGAATAAGGAGT | | |  |
| AH1408 | AGCAGGATCCATGGCAACAATAAAAGATGTAGCGAAACGA | | |  |
| AH1409 | CAGGAAGCTTAGTGATGGTGATGGTGATGACGACGATAGTCGCGGAACGG | | |  |
| AH1411 | GCGCTAGGGGAGAAGTGTAATTCTTTGA | | |  |
| AH1413 | CGCTAGGGGACTCGTGTAATTCTTTGA | | |  |
| AH1414 | GATAGCGCCCGGAAGAGAGTCAATTCAGGGTGGTGAATGTGTCCGGGGATC  CGTCGACCT | | |  |
| AH1415 | AATGAGTGAGCTAACTCACATTAATTGCGTTGCGCTCAGTGTAGGCTGGAGC  TGCTTCG | | |  |
| AH1416 | CAGTGTCGACGGATGGTCGTACCGCCATATCG | | |  |
| AH1417 | CAGGAAGCTTCGCTCACAATTCCACACAACATACGA | | |  |
| AH1418 | AGCAGGATCCATGAAACCAGTAACGCTATACGATGTCGCAGAGT | | |  |
| AH1419 | CAGGAAGCTTAGTGATGGTGATGGTGATGCTGCCCGCTTTCCAGTCGGGAAAC | | |  |
| AH1420 | GTGGCACAACAACTGGCGGG | | |  |
| AH1421 | GCGGTTGGGAATGAAATTCAGCTC | | |  |
| AH1423 | GCGGTTGGGAATCTCATTCAGCTC | | |  |
| AH1424 | GGTAGAACCGTAGGTCGGATAAGA | | |  |
| AH1425 | CGTAATCAATCCAGACCATATGTGTCC | | |  |
| AH1426 | CGATTCATTAATGCAGCTGGCACG | | |  |
| AH1427 | CGACGGCCAGTGAATCTGTAATCA | | |  |
| AH1475 | CGACGGTAACCACCGAGCCAA | | |  |
| AH1476 | GGTGTTTCTCCAACAAGCTTCCATC | | |  |
| AH1495 | CTGTCATGTACGATCCTTGCCC | | |  |
| AH1496 | CCAACGAACCCTGCAGATCATTTATAGG | | |  |
| K5359 | CACATACAACAAGTCCATACATTC | | |  |
| K5360 | CGAGTCCATCATCAGGCACATTA | | |  |
| K5361 | GCAACCGCTGCTTTGGTTGG | | |  |
| K5362 | CGACATCAGCAACACTTTCCG | | |  |
| K5365 | CACCGAAGCTTGCAACAGATATGTTTATACAGGCATCAT | | |  |
| K5366 | GCACCGGGATCCACTGGTAGATTTATCTACAGGTG | | |  |
| K5831 | AGTGCTCGTTTTTCCCTTGA | | |  |
| K5832 | AGCGAAGAACTTTTGCCTCA | | |  |
| K5833 | CTCAGTGGATGAGAAGACAGGAG | | |  |
| K5834 | CCGCAGTAAGAGTACCTAGTACG | | |  |
| K6088 | GCATCATCCCTTACCGTGGTTC | | |  |
| K6089 | GGATCTGCTCTGTGGTGTAGTTCA | | |  |

**References**

1. **Donohue-Rolfe, A, Kondova, I., Oswald, S., Hutto, D., and Tzipori, S.** 2000. *Escherichia coli* O157:H7 strains that express Shiga toxin (Stx) 2 alone are more neurotropic for gnotobiotic piglets than are isotypes producing only Stx1 or both Stx1 and Stx2. J.Infect.Dis. **181**:1825-1829.

2. **Lajoie, MJ, Rovner, A. J., Goodman, D. B., Aerni, H. R., Haimovich, A. D., Kuznetsov, G., Mercer, J. A., Wang, H. H., Carr, P. A., Mosberg, J. A., Rohland, N., Schultz, P. G., Jacobson, J. M., Rinehart, J., Church, G. M., and Isaacs, F. J.** 2013. Genomically recoded organisms expand biological functions. Science **342**:357-360.

3. **Cherepanov, PP and Wackernagel, W.** 1995. Gene disruption in *Escherichia coli*: TcR and KmR cassettes with the option of Flp-catalyzed excision of the antibiotic-resistance determinant. Gene **158**:9-14.

4. **Young, TS, Ahmad, I., Yin, J. A., and Schultz, P. G.** 2010. An enhanced system for unnatural amino acid mutagenesis in *E. coli*. J.Mol.Biol. **395**:361-374.

5. **Datsenko, KA and Wanner, B. L.** 2000. One-step inactivation of chromosomal genes in *Escherichia coli* K-12 using PCR products. Proc.Natl.Acad.Sci.U.S.A **97**:6640-6645.

6. **Stokes, MG, Titball, R. W., Neeson, B. N., Galen, J. E., Walker, N. J., Stagg, A. J., Jenner, D. C., Thwaite, J. E., Nataro, J. P., Baillie, L. W., and Atkins, H. S.** 2007. Oral administration of a *Salmonella enterica*-based vaccine expressing *Bacillus anthracis* protective antigen confers protection against aerosolized *B. anthracis*. Infect.Immun. **75**:1827-1834.

7. **Hansen, AM, Chaerkady, R., Sharma, J., Diaz-Mejia, J. J., Tyagi, N., Renuse, S., Jacob, H. K., Pinto, S. M., Sahasrabuddhe, N. A., Kim, M. S., Delanghe, B., Srinivasan, N., Emili, A., Kaper, J. B., and Pandey, A.** 2013. The *Escherichia coli* phosphotyrosine proteome relates to core pathways and virulence. PLoS.Pathog. **9**:e1003403.
